# Isomerization pathway of a C–C sigma bond in a bis(octaazamacrocycle)dinickel(II) complex activated by deprotonation: a DFT study

**DOI:** 10.1007/s00214-024-03100-5

**Published:** 2024-03-13

**Authors:** Ingrid Jelemenska, Michal Zalibera, Peter Rapta, Anatoly A. Dobrov, Vladimir B. Arion, Lukas Bucinsky

**Affiliations:** 1 Institute of Physical Chemistry and Chemical Physics, Faculty of Chemical and Food Technology, Slovak University of Technology in Bratislava, Radlinského 9, 81237 Bratislava, Slovak Republic; 2 Faculty of Chemistry, Institute of Biophysical Chemistry, University of Vienna, Josef-Holaubek-Platz 2, 1090 Vienna, Austria; 3 Institute of Inorganic Chemistry, University of Vienna, Währinger Strasse 42, 1090 Vienna, Austria

**Keywords:** DFT, Nickel complexes, Isomerization pathway, Anti-syn isomer, Scan, Potential energy surfaces

## Abstract

The anti (**a**) to syn (**s**) isomerization pathway of the deprotonated form of the dimer with two nickel(II) 15-membered octaazamacrocyclic units connected via a carbon–carbon (C–C) σ bond was investigated. For the initial anti (**a**) structure, a deprotonation of one of the bridging (*sp*^3^ hybridized) carbon atoms is suggested to allow for an **a** to **s** geometry twist. A 360° scan around the bridging C–C dihedral angle was performed first to find an intermediate geometry. Subsequently, the isomerization pathway was explored via individual steps using a series of mode redundant geometry optimizations (internal coordinates potential energy surface scans) and geometry relaxations leading to the **s** structure. The prominent geometries (intermediates) of the isomerization pathway are chosen and compared to the **a** and **s** structures, and geometry relaxations of the protonated forms of selected intermediates are considered.

**Supplementary Information:**

The online version contains supplementary material available at 10.1007/s00214-024-03100-5.

## Introduction

Investigation of transition-metal complexes with redox non-innocent ligands has been the subject of great research interest over the last few decades [1–3]. A detailed understanding of the electronic structures [4, 5] of these compounds and their use in studies of stoichiometric reactivity [6–11] and catalytic transformations [12–14] have been reported. Nowadays, the number of studies reporting the use of first-row transition-metal complexes with related ligands in catalytic reactions is increasing [15, 16]. Catalytic activity of nickel(II) complexes was reported in the oxidation of cyclohexane to cyclohexanol and cyclohexanone [17, 18], in the alcohol oxidation [15] and in the electrocatalytic reduction [19, 20] studies. Furthermore, the redox activity of nickel compounds was investigated in the perspective of the carbon dioxide reduction [21–25], water oxidation [26, 27] as well as photoredox catalytic C–C bond formation reactions [28–30]. Nickel(II) complexes with S-substituted isothiosemicarbazides (H_2_NNC(SR)NH_2_) and isothiosemicarbazones (R_1_R_2_C=NNC(SR)NH_2_) are known for their redox properties. The isothiocarbohydrazides (NH_2_NC(SR)NHNH_2_) and the isothiocarbohydrazones (R_1_R_2_C=NNC(SR)NHNH_2_) are closely related derivatives that exhibit redox non-innocent behavior [31, 32]. Recently, we have reported several new nickel(II) complexes with 14- and 15-membered octaazamacrocyclic ligands [33]. This study reported the structure and spectroscopic properties of 1e-oxidized and 1e-reduced species. Furthermore, the ability to catalyze microwave-assisted solvent-free oxidation of cyclohexane by tert-butyl hydroperoxide (TBHP) to an industrially significant mixture of cyclohexanol and cyclohexanone (that is, A/K oil) has also been reported [33].

The paramagnetic intermediates and final diamagnetic products generated after cathodic reduction of monomeric nickel(II) 15-membered octaazamacrocyclic complexes were studied by applying a collaboration of electrochemical and spectroelectrochemical techniques, including cyclic voltammetry, in situ EPR and EPR/UV–vis–NIR spectroelectrochemistry [34], as well as ex situ NMR and MS spectroelectrochemistry. Density functional theory (DFT) calculations were performed to further support the assignment of species generated after reduction [35]. In a follow-up work, the analysis of the relationship between the electronic structure of the 15-membered octaazamacrocyclic nickel complexes and their affinity for CO_2_ binding was addressed [33, 35, 36].

**Fig. 1:**
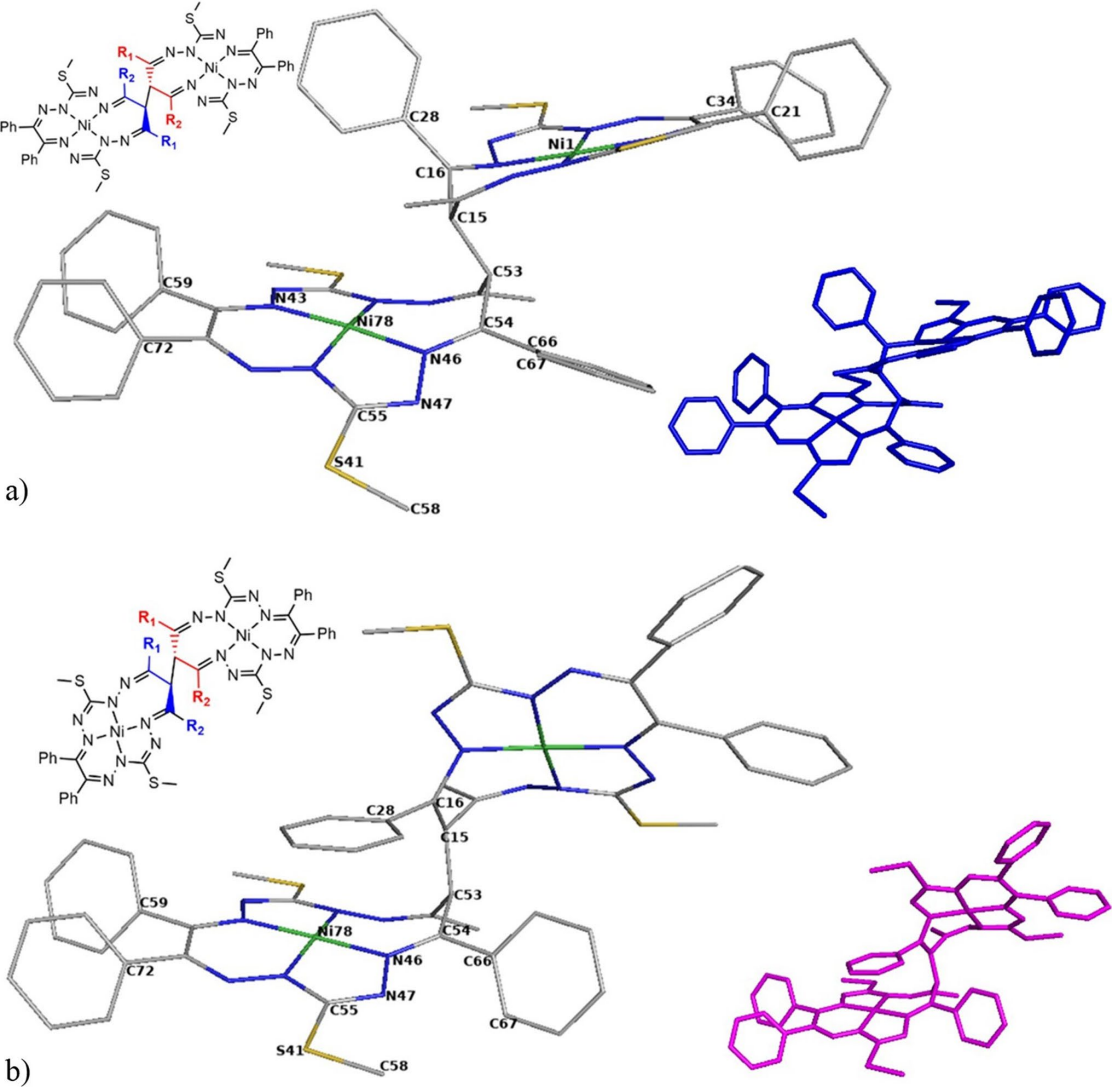
The skeleton of the studied **a**) **4a** and **b**) **4s** structure with atom labeling scheme as used within this study (R_1_ = Me; R_2_ = Ph) and skeleton of the anti (**a**) structure-blue color and syn (**s**) structure-magenta color. H-atoms are not shown

Although the number of mononuclear complexes with redox non-innocent ligands is large and increases permanently, reports on dinuclear metal complexes with this kind of ligands are still scarce [37–39]. Quite recently, Dobrov et al. [40] described the synthesis of diastereomeric dinickel(II) complexes (denoted as **4**–**6**) with new bis(octaazamacrocyclic) ligands (denoted as **1**–**3**), where Fig. 1 shows the anti (**a**) and syn (**s**) forms of **4** that will be further explored in this study. Geometric **a** and **s** isomers (enantiomers) were separated chromatographically, isolated, and characterized by analytical and spectroscopic techniques, as well as by single-crystal X-ray diffraction (SC-XRD) (**4a** and **4s**, **5s**, and **6a**). The isomerization kinetics of **5** (the conversion of **5a** to **5s**) was investigated in chloroform by ^1^H NMR spectroscopy (it was monitored at five different temperatures from 20 °C to 50 °C), and the thermodynamics was compared with the theoretical DFT results [40]. The anti-syn isomerization (epimerization) of **5** was found to be a clean conversion from 5a to 5s, with the activation barrier of Δ*H*^≠^  = 114 ± 1 kJ mol^–1^ and the activation entropy of Δ*S*^≠^  = 13 ± 3 J K^–1^ mol^–1^ according to the Eyring equation [40]. It was shown that the half time of conversion from **5a** (with a starting concentration of approximately x = 92%) to **5s** was approximately 14 and 56 h at 50 and 40 °C, respectively (half times of conversions at 30 and 21 °C extrapolated from the kinetic model were 278 and 1111 h, respectively) as shown in Figure S5 in the Electronic Supplementary Information of Dobrov et al. [40]. Complex **4a** was identified as the (*R*,*S*) or (*S*,*R*)-meso-isomer, where both form the same structure due to the presence of two identical monomeric units and an inversion center at the midpoint of the C15–C53 bridge (see Fig. 1). On the other hand, the complex **4s** crystallized in a centrosymmetric triclinic space group $P\overline{1 }$ (inversion center) as a racemic mixture of the (*R*,*R*)- and (*S*,*S*)-enantiomers [40]. It is worth noting that **4** is the only complex with crystal structures of both diastereomers **4a** and **4s** reported in the original reference [40].

In this paper, we extend the experimental study of Dobrov et al. [40] with a theoretical isomerization pathway consideration of an anti (**a**) dinuclear Ni(II) complex conformer, which is energetically less favored, to the energetically preferred syn (**s**) one. Therefore, the initial structure in this anti-syn isomerization pathway study is the anti (**a**) conformer. To allow the flexibility to undergo isomerization, we assume that one of the bridging *sp*^3^ hybridized carbons (C15) is deprotonated, see Fig. 1. For this initial structure of the anti conformer **a**, a scan of the bridging dihedral angle C54–C53–C15–C16 was performed in the range from 0 to 360° and a step size of 10°. Subsequently, the anti-syn isomerization pathway was explored using a potential energy surface (PES) scan with mode redundant (active/frozen) internal coordinate settings. Finally, selected C15 back-protonated intermediate structures were relaxed to report their anti-intermediate-syn preference.

### Computational details

Standard all-electron (C, H, N, S, Ni) B3LYP/6-31G* [41–48] geometry optimizations in the preferred singlet spin state were performed using the Gaussian09 [49] program package. The stability of the optimized structures was confirmed by vibrational analysis (no imaginary frequencies). In addition, B3LYP calculations with the 6-311G** basis set [27–34] have been taken into account as well as the implicit integral equation formalism polarizable continuum model (IEFPCM) [50, 51] of CH_2_Cl_2_ to compare with the energetics of the in vacuo B3LYP/6-31G* calculations. Unless otherwise stated, in vacuo B3LYP/6-31G* calculations were reported. The potential energy surface scans were done with the opt(redundant) regime of Gaussian09, i.e., chosen internal coordinates are changed in a predefined sequence of steps, and kept frozen (constant) during the geometry optimization. Relative total SCF energies are considered in the potential energy surface scans, since a vast majority of these points are not minima on the potential energy surface with a frequency calculation (zero point correction) being biased due to nonzero gradients. Relative enthalpies and Gibbs free energies are presented for protonated and deprotonated structures that are minima on the potential energy surface. Atoms of internal coordinates which were scanned or frozen (see below) are labeled in Fig. 1. The IQmol package [52] has been used for the visualization of the obtained structures. The overlap of selected structures was visualized with the PyMOL package [53].

## Results

According to the experimental findings, the anti (**a**) structure is energetically less favored than the syn (**s**) structure for complex **4**. Therefore, the **a** isomer is chosen as the initial structure in this study of the anti-syn isomerization pathway. Additionally, to allow for the necessary flexibility, the C15 bridging sp^3^ hybridized carbon is deprotonated. The acidity of protons on these carbon atoms was found in monomeric species (the CH_2_ group of the monomer can donate a proton) [33]. In addition, the option of a tautomeric form of the dimeric species with a proton transfer from the carbon bridging atom to a nitrogen atom of the macrocycle has been considered. It was found that the total SCF energy difference between these species (123.6 kJ·mol^–1^) is already comparable to the experimentally estimated energy barrier (117 ± 1 kJ·mol^–1^) [40]. Thus, carbon C15 is considered as *sp*^2^ hybridized (lack of proton) in this study to allow exploration of the anti-syn isomerization pathway. The proton transfer B3LYP/6-31G* reaction total SCF energy, enthalpy and Gibbs free energy (**a**_H_ + OH^−^ → **a** + H_2_O) is –336, –300, and –306 kJ mol^−1^, respectively. The B3LYP/6-31G* total SCF energy, enthalpy, and Gibbs free energy differences between the deprotonated forms **a** and **s** obtained from the geometry optimization of crystal structure geometries are − 0.367, 0.974 and 0.110 kJ mol^−1^, respectively. In the case of protonated forms, the **a**_**H**_-**s**_**H**_ B3LYP/6-31G* total SCF energy, enthalpy, and Gibbs free energy differences are − 5.413, − 4.826 and 0.958 kJ mol^−1^, respectively. In addition, when solvent effects become considered (IEFPCM model of CH_2_Cl_2_), the B3LYP/6-31G* total SCF energy, enthalpy, and Gibbs free energy differences between the deprotonated (protonated) forms **a** and **s** are − 0.929, 3.229, and − 0.297 (− 5.464, − 7.312, and − 6.073) kJ mol^−1^, respectively. Implicit IEFPCM model of CH_2_Cl_2_ B3LYP/6-311G** total SCF energy, enthalpy, and Gibbs free energy differences between the deprotonated (protonated) forms **a** and **s** are − 0.502, 0.029, and 0.000 (− 4.072, − 4.742, and − 0.004) kJ mol^−1^, respectively. Hence, the *in vacuo* B3LYP/6-31G* calculations lead to an acceptable agreement with a higher quality basis set and implicit solvent effects [54]. It needs to be also mentioned that the accuracy of DFT functionals, where B3LYP is being the part of most of these studies, regarding thermochemistry, is at a limit of 5 kJ mol^−1^, and often is reported to be worse than 15 kJ mol^−1^ [54–56]. Below, the labels **a** and **s** denote the deprotonated forms of **4**, while the protonated forms are labeled as **a**_**H**_ and **s**_**H**_.

### Scan of the bridging dihedral angle

**Fig. 2:**
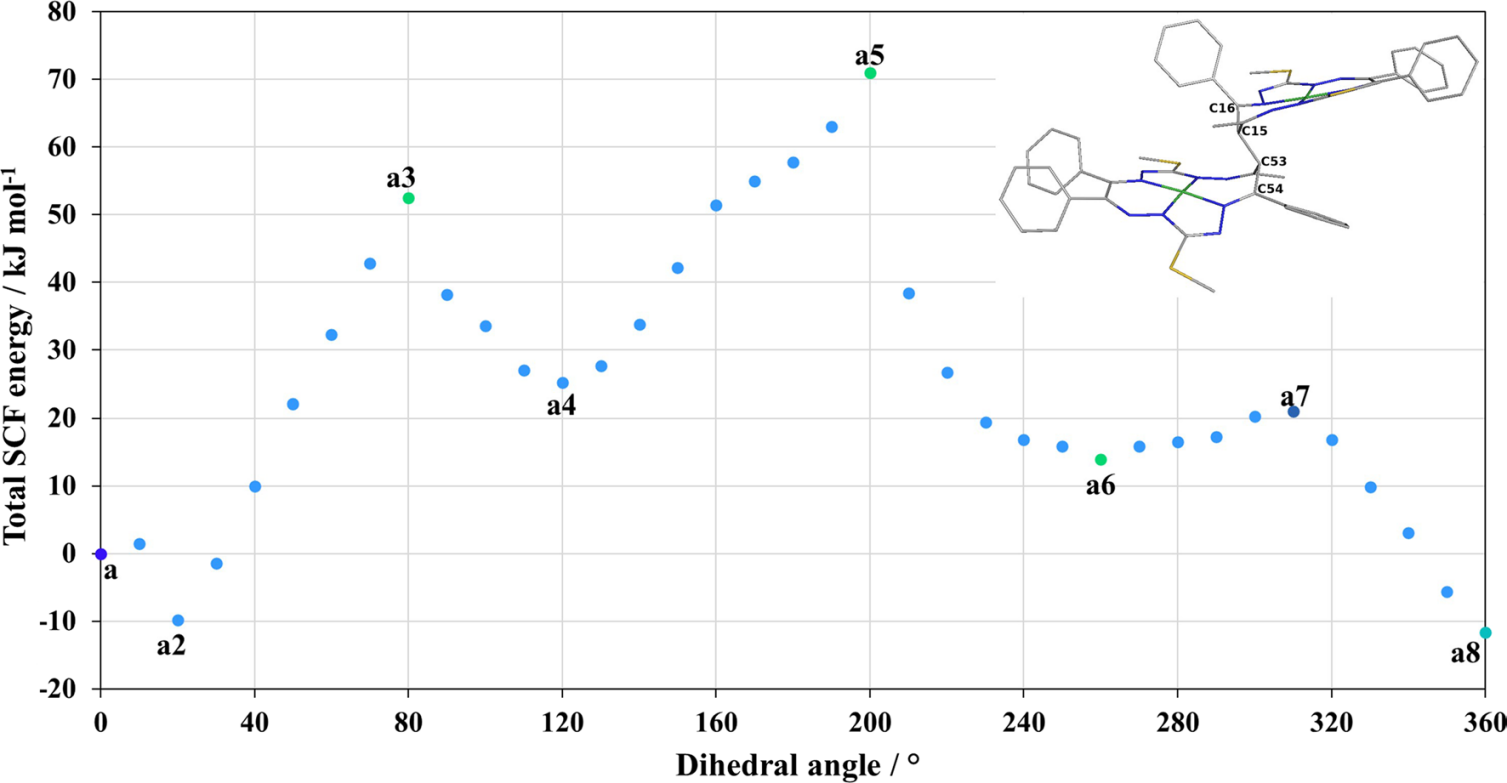
Potential energy surface scan of the bridging dihedral angle for atoms C54–C53–C15–C16 from 0 to 360° for the initial structure **a**

Prior to the actual anti-syn isomerization study, a relaxed scan of the bridging dihedral angle of C54–C53–C15–C16 atoms from 0° to 360° was performed for the initial deprotonated structure **a**, see in Fig. 2. The found minima and maxima on the PES are highlighted as **a1**–**a8**, their total B3LYP/6-31G* energies are compiled in Table S1. In the scan, we found a minimum (**a2**), which is to be related to the relaxation of one of the phenyl rings (C66; see Fig. 1) to an energetically preferred orientation. The structure **a2** is below the initial structure by − 9.796 kJ mol^−1^ (where **a** = **a1**). After the full scan, the new relaxed structure **a8** is 11.635 kJ mol^−1^ below the initial structure **a**. The difference in the structures **a** and **a8** is shown in Fig. 4a, here we can see the perpendicular orientations of R_2_ phenyl rings on C54 (note that the **a2** and **a8** structures are readily the same, not shown). Furthermore, there are the maxima on the dihedral angle scan, **a3**, **a5,** and **a7** which are 52.529 (64.164), 70.960 (82.595), 21.043 (32.678) kJ mol^−1^, respectively, above the initial structure **a** (**a8**). From the PES scan, it can be concluded that structure **a8** can be considered the starting point of the anti-syn isomerization of the studied Ni macrocyclic dimer (let us emphasize again that we are considering the deprotonated form). According to the dihedral angle C54–C53–C15–C16 scan, the structure **a7** will be considered as the first intermediate geometry of this anti-syn isomerization pathway study, according to the lower height of the barrier comparing to **a3**.

In addition, dihedral angle scans of C-SMe and phenyl groups are shown in Supplementary Materials (see Figs. S1 and S2). In the case of the scan of N46–C54–C66–C67 (phenyl group), the maximum on this PES is below 24.6 kJ mol^−1^, see Fig. S1. The total SCF energy difference between the first and last geometry of the scan is − 0.354 kJ mol^−1^. The found maxima of the scan of the dihedral angle N47-C55-S41-C58 (C-SMe group) are below 28.8 kJ mol^−1^ and 31.82 kJ mol^−1^, see Fig. S2.

### Scan of the potential energy surface: the anti-syn isomerization pathway

**Fig. 3:**
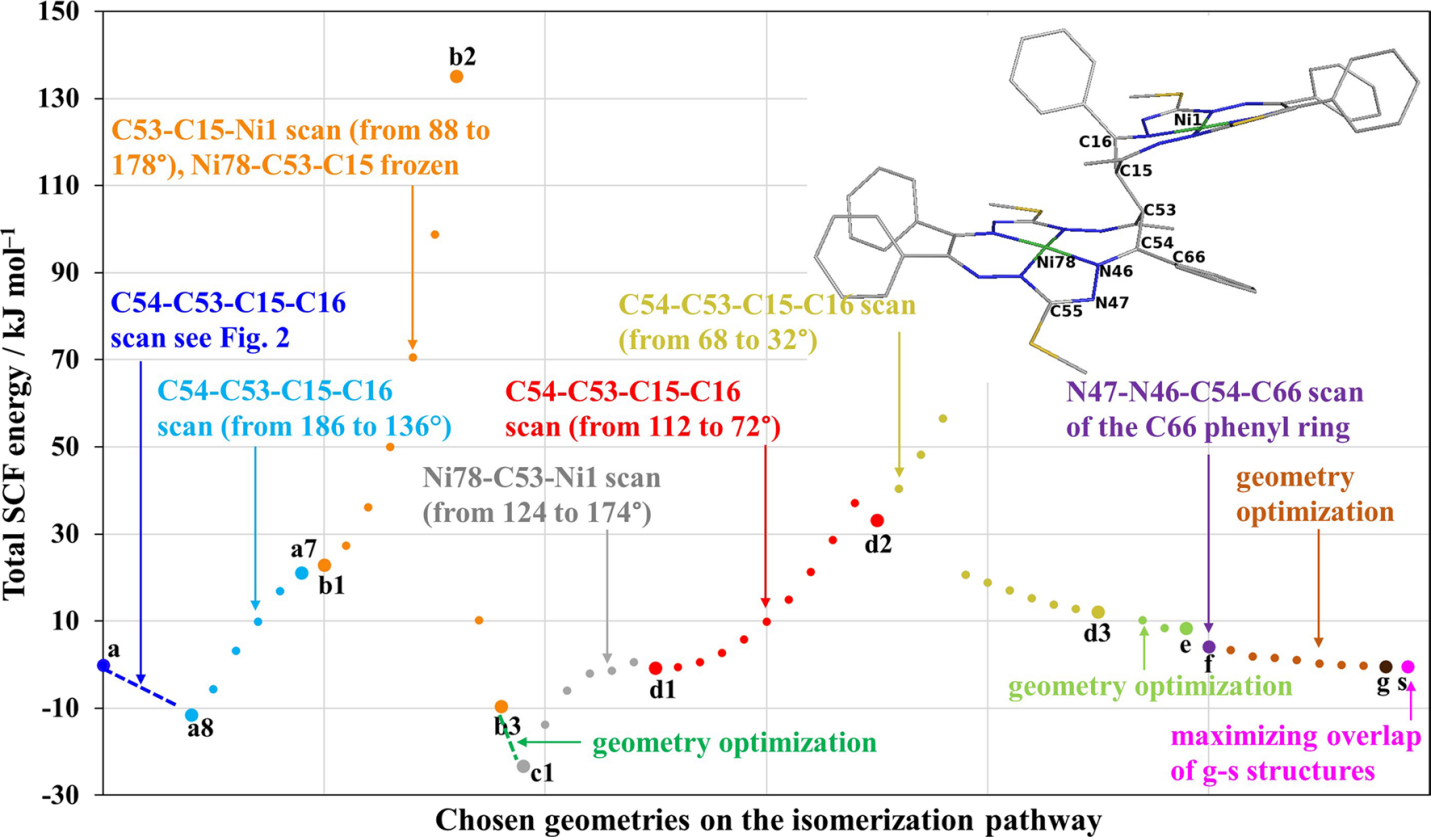
Constrained potential energy surface scan. On the independent axis (*x*) is the anti-syn isomerisation pathway and on the dependent axis (*y*) is the total SCF energy difference in kJ mol^–1^ relative to **a**. Details of the isomerization pathway (scanned and frozen coordinates) are presented in the particular color

**Fig. 4:**
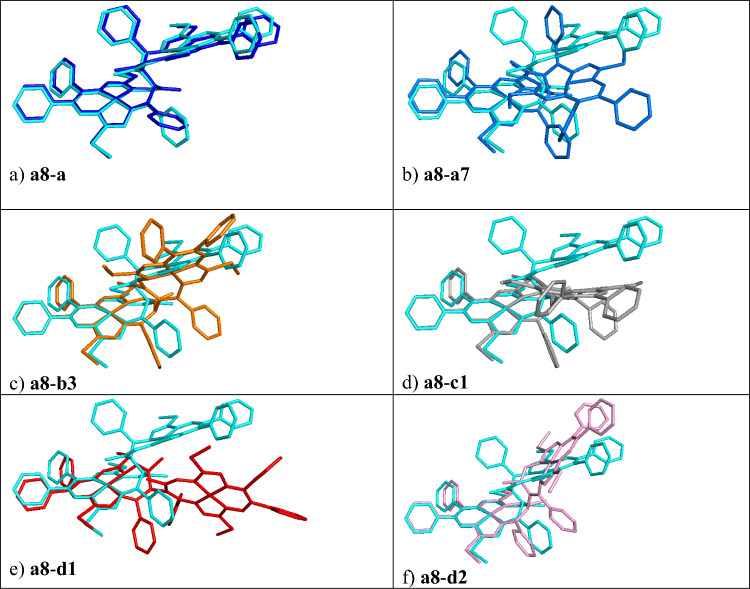
Overlap of initial structure **a8** (cyan) with selected structures of the isomerization pathway

**Fig. 5:**
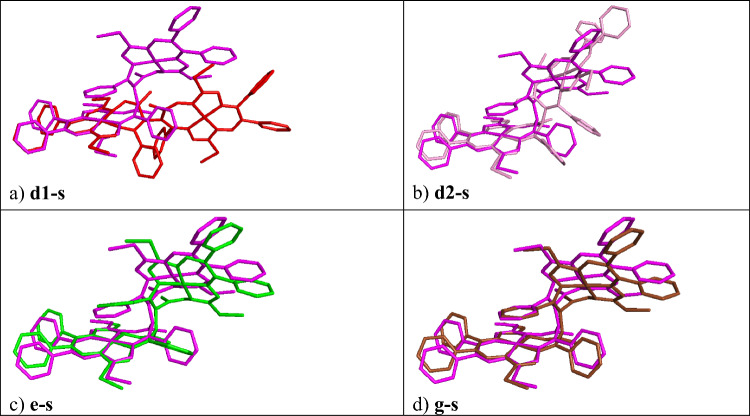
Overlap of selected geometries with the final **s** (violet) isomer

As already mentioned, the obtained geometry **a8** is the start for the PES exploitation to suggest an anti-syn isomerization pathway. The found isomerization pathway with the description of the individual steps/intermediates (**a**–**g**) is shown in Fig. 3, including the optimization details, i.e., active and frozen (PES scan, non-redundant) coordinates, or geometry relaxation (ordinary or constrained geometry optimization). The Cartesian coordinates of optimized geometries (**a**–**s**) are accounted for in Supplementary Materials (see Tables S3–S15). The differences (overlaps) of succeeding structures during the PES exploitation with the initial structure **a8** are shown in Fig. 4, and the overlap of the selected geometries with the final syn (**s**) structure is compiled in Fig. 5, to show the additional changes in geometry (e.g., phenyl and C-SMe groups orientation).

The difference in the backbone of structures **a**–**a8** during the relaxation of the crystal structure like geometry **a** is due to the C66 phenyl ring reorientation, see Fig. 4a (a8 is the cyan reference structure). The difference of **a8**–**a7** structures during the C54–C53–C15–C16 dihedral angle scan, from 135.8 to 185.8°, step = 10°, #steps = 5 is shown in Fig. 4b (**a7** is the dark blue structure).

The geometry of structure **a7** was then used for a further C53–C15–Ni1 angle PES scan keeping the Ni78-C53-C15 angle frozen, see orange points in Fig. 3. The overlap of structures **a8**-**b3** is shown in Fig. 4c. The PES maximum of **b2** is 135.0 (146.7) kJ mol^–1^, above the initial structure **a** (**a8**), see Table S1 and Fig. 3.

The overlap of the initial structures **a8** and **c1** (see gray structure in Fig. 4d) shows the difference due to the relaxation of the angle C53–C15–Ni1 which was frozen in **b** (orange points in Fig. 3). The C53–C15–Ni1 angle changes from 169° in **b3** to 155° in **c1**. The energy difference between **b3**–**c1** is –13.608 kJ·mol^–1^. Surprisingly, the energetically preferred structure for the deprotonated form is **c1**. The existence of the **c1** minimum in the PES of the deprotonated forms is to be considered an important intermediate on the isomerization pathway. The protonated form of **c1**_**H**_ is not energetically preferred compared to **a**_**H**_ and **s**_**H**_, see Table 1 and in the next section devoted to geometry relaxations of the back-protonated forms.

**Table 1:** B3LYP/6-31G* total SCF energies and relative total SCF energies, relative enthalpies, and relative Gibbs free energies for protonated intermediates, where ^1^[**a**_**H**_]^0^ means singlet spin state and neutral form and is chosen as the reference structure

B3LYP	*E* [hartree]	Δ*E* [kJ/mol]	Δ*H* [kJ/mol]	Δ*G* [kJ/mol]	
^1^[**a**_**H**_]^0^	− 7649.06490	0.000	0.000	0.000	anti
^1^[**a2**_**H**_]^0^	− 7649.06845	− 9.331	0.483	− 8.050	anti
^1^[**a8**_**H**_]^0^	− 7649.06844	− 9.310	0.554	− 7.953	anti
^1^[**b1**_**H**_]^0^	− 7649.06846	–9.336	0.882	− 8.021	anti
^1^[**b3**_**H**_]^0^	− 7649.05011	38.857	48.034	39.393	anti (76°)
^1^[**c1**_**H**_]^0^	− 7649.05011	38.851	48.028	39.327	anti (76°)
^1^[**d1**_**H**_]^0^	− 7649.00280	163.052	169.613	161.487	cross intermediate
^1^[**d2**_**H**_]^0^	− 7649.04282	57.971	61.429	57.564	syn (150°)
^1^[**d3**_**H**_]^0^	− 7649.06499	− 0.220	5.768	0.312	syn
^1^[**e**_**H**_]^0^	− 7649.06500	− 0.237	6.595	0.391	syn
^1^[**f**_**H**_]^0^	− 7649.06836	− 9.069	− 1.050	− 7.984	syn
^1^[**g**_**H**_]^0^	− 7649.06837	− 9.100	− 0.827	− 7.979	syn
^1^[**s**_**H**_]^0^	− 7649.06697	− 5.413	0.958	− 4.826	syn

The gray part (**c1**–**d1**) of the isomerization pathway was based on the scan of the Ni78-C53-Ni1 angle (see Fig. 3). The energy difference between **c1** and **d1** is –22.460 kJ·mol^–1^ (**d1** is above **c1**, see Table S1, Fig. 3). In this part, the actual isomerization from anti (**a**) to syn (**s**) happens. The structure **d1** (see the red structure in Figs. 4e and 5a) is by 0.844 (10.791) kJ mol^–1^ below the initial structure **a** (**a8**).

The red and yellow part of the isomerization pathway (see Fig. 3) continues with the C54–C53–C15–C16 dihedral angle PES scan from 112° to 72° (68° to 32°) for **d1**–**d2** (**d2**–**d3**) structures, respectively, see Fig. 3. The PES maximum of the yellow part (not labeled) is 56.569 (68.203) kJ mol^–1^ above the initial structure **a** (**a8**). The overlaps of **d2** (pink structure) with **a8** and **s** are shown in Figs. 4f and 5b, respectively. After the **d** scan, the **d3** geometry was used as the input for a geometry optimization/relaxation (see structure **e** in Fig. 3). The overlap of the structures **d3-s** is essentially the same as for **e**-**s** (green structure) shown in Fig. 5c, i.e., the major difference between **d3** and the **s** isomer is the orientation of the C66 phenyl ring. The energy difference between structure **a** and **e** is 8.331 kJ mol^–1^. Thus, the next PES scan (**e** to **f**) was performed for the rotation of the C66 phenyl group of **e** (see Fig. 3).

The final geometry optimization was performed for the structure **g** (see the brown part in Fig. 3). The close overlap of **g** (brown structure) with the **s** isomer is shown in Fig. 5d. The structure **g** is –0.359 kJ mol^–1^ below the initial structure **a** (respectively, the structure **g** is 11.275 kJ mol^–1^ above **a8**) in total SCF energy. Note that **s** is by –0.367 kJ mol^–1^ below **a**, therefore, **g** should be considered energetically degenerate with **s**. Relative enthalpies and Gibbs free energies are provided for selected relaxed deprotonated structures in Table S2.

### Stability and relaxation of the back-protonated intermediates

As a final step within this study, the relaxation of the back-protonated forms of selected intermediate structures was performed. A proton has been added to the carbon C15 atom being *sp*^3^sp^3^hybridized in the (back-)protonated forms.

The protonated form of **a** is denoted as **a**_**H**_. The intermediate structure **b1**_**H**_ relaxes to the anti isomer. This structure defines the actual energy minimum of the protonated **a** forms. Relaxed intermediate structures of **b3**_**H**_ and **c1**_**H**_ are energetical and geometric equivalents of an anti isomer with a bridging dihedral angle C54–C53–C15–C16 of 76° (that is, an **a6**_**H**_ perpendicular like structure of Fig. 2). The structure **d1**_**H**_ is the anti to syn cross intermediate structure (the bridging dihedral angle C54–C53–C15–C16 is 78° and the angle Ni78–C53–Ni1 is 173°). The remaining structures (from **d2**_**H**_ to **g**_**H**_) relaxed to the geometry of a syn isomer (with **d2**_**H**_ being an **a4**_**H**_ perpendicular like structure of Fig. 2). The protonated syn structure has two possible variants with respect to chirality of the bridging carbons. Actually, the original structures were found as the racemic mixtures of *S*,*S* and *R,R* and the isomerization pathway resulted in the *R*,*R* isomer. Furthermore, the difference in the preference of the ^1^[**a**_**H**_]^0^ and ^1^[**s**_**H**_]^0^ structures hints on the complexity of the PES structure with respect to the presence of several local minima, exploitation of which is beyond the scope of this work. Still, the activation barrier of the deprotonated forms is in agreement with the experimental kinetics reported for compound **5** in the original study. It can be assumed that the enforced (mode redundant) geometry PES scans lead to an overestimated energy barrier in Fig. 3, structure **b2**. Furthermore, deprotonation needs to occur, to activate the suggested isomerization pathway. Another option is the tautomerization of the proton to any of the nitrogens on the ligand macrocycles, as discussed in the original reference [40], to activate the mechanism of the carbon–carbon isomerization pathway. The found anti  and syn pronated forms are to be qualified degenerate, and albeit the crystal structure relaxed geometries led to the thermodynamic preference of the syn structure, the degenerate situation is acceptable with respect to the accuracy of DFT calculations [54–56].

## Conclusions

The anti-syn isomerization pathway of the deprotonated form of dinickel(II) complexes with C–C coupled 15-membered octaazamacrocyclic ligand was successfully investigated theoretically. The initial anti structure **a8** and final structure **s**, were found a local minima in comparison with the found **c1** structure. The actual isomerization from anti (**a**) to syn (**s**) is found to occur in the part **c1**–**d1** of the followed pathway.

Finally, the relaxation of the back-protonated forms of selected intermediate structures of the isomerization pathway was performed. According to our results, structures **b1**_**H**_, **b3**_**H**_ and **c1**_**H**_ are relaxing back to the anti isomer. The structure **d1**_**H**_**d**_**1H**_is the anti to syn cross intermediate structure. While structures from **d2**_**H**_ to **g**_**H**_ relaxed to the geometry of the syn isomer. The protonated syn structure has two possible chiral variants, and the isomerization pathway followed herein led to the *R*,*R* enantiomer, note that both crystal types, i.e., (*R,R*) and (*S,S*), were found in the original study [40]. A further issue is the energy barrier height, which is found to be overestimated in comparison with the experimentally derived value. In this place, we need to accept that the guided PES scan was unable to find the optimal path when crossing from the **b1** to the **b3** intermediate. The found back-protonated structures are found to be energetically degenerate which is acceptable with respect to the accuracy of DFT calculations [54–56].

## Supplementary Materials

Figures of dihedral angle scan of phenyl and C-SMe groups for the **s** structure; Table of total and relative SCF energies for selected deprotonated structures; Table of relative enthalpies and Gibbs free energies of selected relaxed deprotonated structures; and the Cartesian coordinates of selected geometries (**a**-**s**) are provided in a docx file.

### Supplementary Information

Supplementary file 1
