# Supplementary material for: Isomerization pathway of a C–C sigma bond in a bis(octaazamacrocycle)dinickel(II) complex activated by deprotonation: a DFT study
Source: Theor Chem Acc. 2024 Mar 13;143(4):26. doi: 10.1007/s00214-024-03100-5 (PMC10937780; doi:10.1007/s00214-024-03100-5)
Supplement: Supplementary file 1 [file 214_2024_3100_MOESM1_ESM.docx]

**Isomerization pathway of a C-C sigma bond in a bis(octaazamacrocycle)dinickel(II) complex activated by deprotonation - a DFT study**

**(Supplementary Materials)**

Ingrid Jelemenská,^#^, Michal Zalibera,^#^, Peter Rapta,^#^, Anatoly A. Dobrov,^≠^, Vladimir B. Arion,^†^, Lukáš Bučinský,^#^

^#^Institute of Physical Chemistry and Chemical Physics, Faculty of Chemical and Food Technology, Slovak University of Technology in Bratislava, Radlinského 9, SK-81237 Bratislava, Slovak Republic

^≠^University of Vienna, Faculty of Chemistry, Institute of Biophysical Chemistry, Josef-Holaubek-Platz 2, 1090 Wien, Austria

^†^University of Vienna, Institute of Inorganic Chemistry, Währinger Strasse 42, A-1090 Vienna, Austria

**Content**

**Fig. S1**. Dihedral angle scan of the phenyl group for the **s** structure **S2**

**Fig. S2**. Dihedral angle scan of the C-SMe group for the **s** structure **S2**

**Table S1**. Total and relative SCF energies for selected deprotonated structures**S3**

**Table S2**. Relative total SCF energies, relative enthalpies, and relative Gibbs free energies for relaxed deprotonated structures**S3**

**Table S3**. Cartesian coordinates of geometry **aS4**

**Table S4**. Cartesian coordinates of geometry **a8S7**

**Table S5**. Cartesian coordinates of geometry **a7S10**

**Table S6**. Cartesian coordinates of geometry **b1S13**

**Table S7**. Cartesian coordinates of geometry **b2S16**

**Table S8**. Cartesian coordinates of geometry **b3S19**

**Table S9**. Cartesian coordinates of geometry **c1S22**

**Table S10**. Cartesian coordinates of geometry **d1S25**

**Table S11**. Cartesian coordinates of geometry **d2S28**

**Table S12**. Cartesian coordinates of geometry **d3S31**

**Table S13**. Cartesian coordinates of geometry **eS34**

**Table S14**. Cartesian coordinates of geometry **gS37**

**Table S15**. Cartesian coordinates of geometry **sS40**


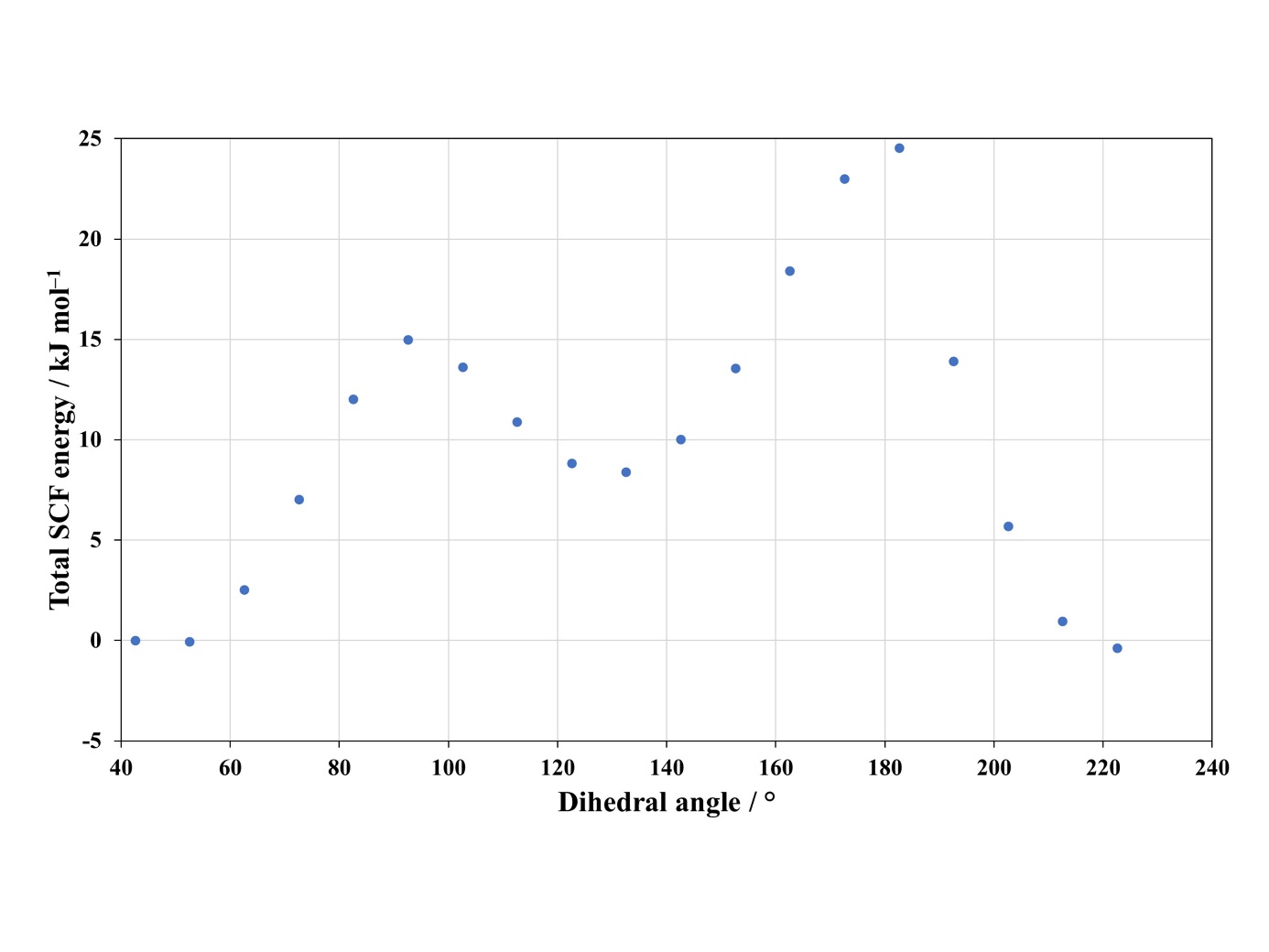


**Fig. S1**. Dihedral angle scan of the phenyl group (atoms N46-C54-C66-C67) for the **s** structure.


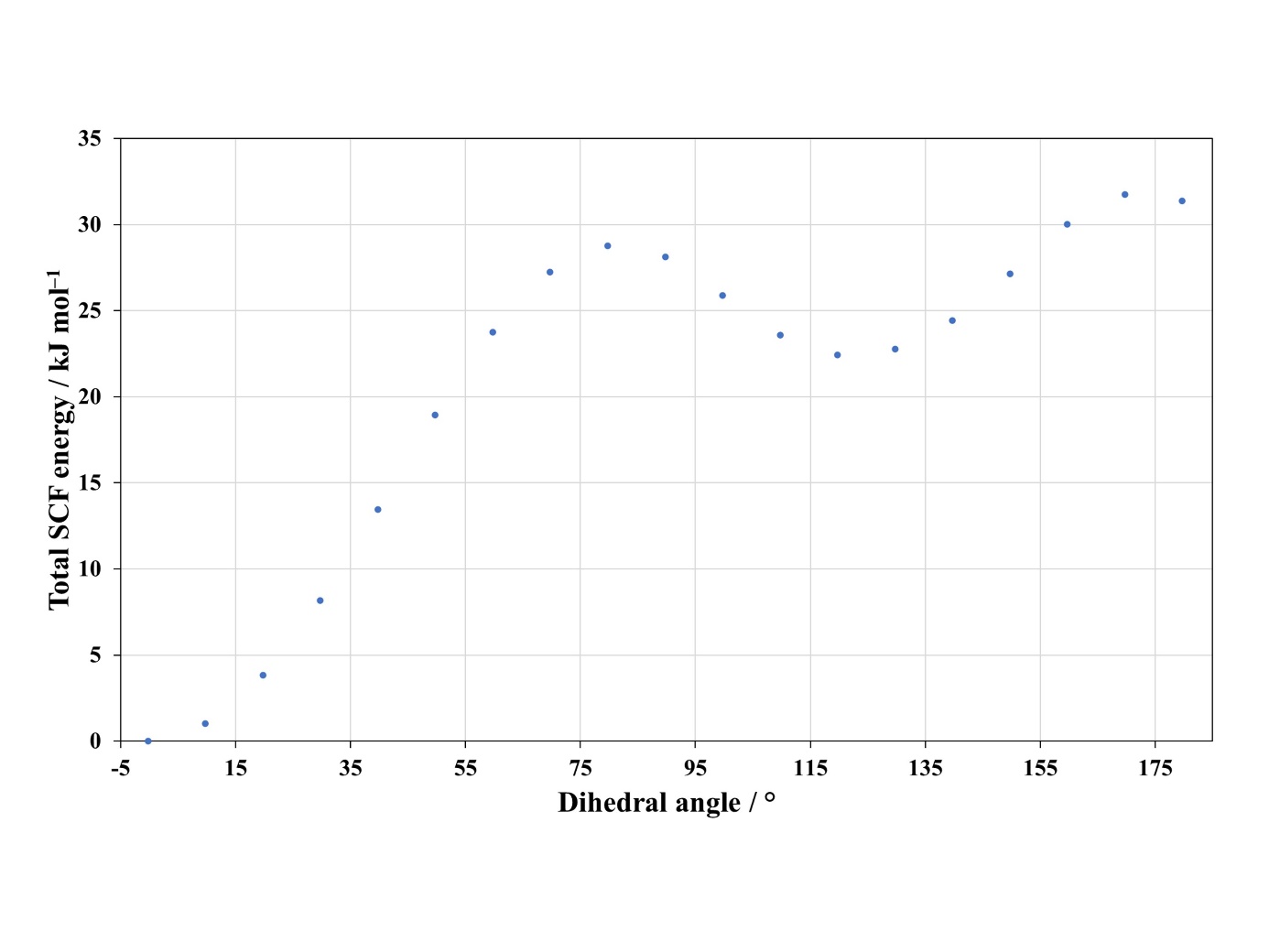


**Fig. S2**. Dihedral angle scan of the C-SMe group (atoms N47-C55-S41-C58) for the **s** structure.

**Table S1**. B3LYP/6-31G* total and relative SCF energies (with ^1^[**a**]^-^ taken as the reference) for selected deprotonated structures.

| B3LYP | E [hartree] | ΔE [kJ/mol] |
| --- | --- | --- |
| ^1^[**a**]^-^ | –7648.50270 | 0.000 |
| ^1^[**a2**]^-^ | –7648.50643 | –9.796 |
| ^1^[**a3**]^-^ | –7648.48269 | 52.529 |
| ^1^[**a4**]^-^ | –7648.49309 | 25.217 |
| ^1^[**a5**]^-^ | –7648.47567 | 70.960 |
| ^1^[**a6**]^-^ | –7648.49740 | 13.897 |
| ^1^[**a7**]^-^ | –7648.49468 | 21.043 |
| ^1^[**a8**]^-^ | –7648.50713 | –11.635 |
| ^1^[**b1**]^-^ | –7648.49398 | 22.882 |
| ^1^[**b2**]^-^ | –7648.45124 | 135.089 |
| ^1^[**b3**]^-^ | –7648.50639 | –9.696 |
| ^1^[**c1**]^-^ | –7648.51157 | –23.304 |
| ^1^[**d1**]^-^ | –7648.50302 | –0.844 |
| ^1^[**d2**]^-^ | –7648.49009 | 33.090 |
| ^1^[**d3**]^-^ | –7648.49811 | 12.041 |
| **^1^[e]^-^** | –7648.49952 | 8.331 |
| **^1^[f]^-^** | –7648.50111 | 4.174 |
| **^1^[g]^-^** | –7648.50283 | –0.359 |
| **^1^[s]^-^** | –7648.50284 | –0.367 |

Table **S2**. B3LYP/6-31G* relative total SCF energies, relative enthalpies, and relative Gibbs free energies for minima on the PES in Fig.3 for selected deprotonated structures (or geometries are relaxed).

| B3LYP | ΔE [kJ/mol] | ΔH [kJ/mol] | ΔG [kJ/mol] |
| --- | --- | --- | --- |
| ^1^[**a**]^-^ | 0.000 | 0.000 | 0.000 |
| ^1^[**a8**]^-^ | -13.407 | -13.078 | -9.486 |
| ^1^[**b2**]^-^ | 135.089 | 133.606 | 131.976 |
| ^1^[**c1**]^-^ | -26.998 | -25.375 | -28.523 |
| ^1^[**d2**]^-^ | 33.090 | 33.388 | 33.790 |
| ^1^[**s**]^-^ | -0.367 | 0.974 | 1.100 |

**Table S3**. Cartesian coordinates of optimized geometry **a**.

opt b3lyp/6-31g* freq=OK charge=-1 multiplicity =1

127

Ni -4.198705126 3.668070405 2.713575421

S -0.889303517 5.974933427 4.525515155

S -7.947024538 1.456150192 2.145633668

N -4.512801367 5.423995312 3.201917638

N -3.455813967 6.090593994 3.786393201

N -2.374164289 4.088763294 3.245501409

N -1.137012699 3.499224651 3.418249491

N -4.070055607 1.871856721 2.203751407

N -5.254864470 1.164429879 2.207460980

N -5.983063228 3.331475642 2.520434429

N -7.022621487 4.130187793 2.520121843

C -5.674010692 6.084032836 3.164660745

C -2.384601105 5.335479058 3.791716441

C -0.791799139 2.383417322 2.859150783

C -1.758446355 1.725329872 1.916330016

C -2.901067604 1.256631575 2.554885332

C -6.254899237 1.980199377 2.299044006

C -6.892009313 5.431273205 2.758119891

C -1.493673383 7.615989065 5.069105330

C -7.650599431 -0.330495179 1.891985056

C -5.715295052 7.495984322 3.663002087

C -4.995026035 8.512900555 3.016514755

C -5.053690993 9.827977845 3.473155479

C -5.822605462 10.149631659 4.596008225

C -6.532156524 9.144565579 5.253299465

C -6.480759195 7.828923218 4.787710625

C 0.566897597 1.847754888 3.220294353

C -2.835408392 0.286655970 3.672552377

C -1.880466556 -0.744635181 3.655482970

C -1.787354271 -1.652065203 4.711588114

C -2.663067877 -1.558734340 5.795048240

C -3.630834820 -0.548967251 5.814416669

C -3.718650720 0.362577567 4.765067896

C -8.180670217 6.173028024 2.605489177

C -8.269280799 7.402862440 1.931595625

C -9.497162832 8.042516228 1.759522760

C -10.670112201 7.465117547 2.248833261

C -10.599821823 6.234759521 2.907359556

C -9.372711354 5.597380296 3.081352954

S -3.730841108 -1.987331382 -1.060431231

S 4.085383008 1.352853436 -0.651145618

N -0.052640426 -1.845578618 0.167049166

N -1.236288250 -2.435366538 -0.175280522

N -1.813460770 -0.197701837 -0.403375426

N -2.805848861 0.639778499 -0.850376541

N 0.240616754 1.864836477 -0.245648720

N 1.545476227 2.218030879 -0.628337895

N 1.822953675 0.000000000 0.000000000

N 2.709349660 -0.925168515 0.343742618

C 0.947687576 -2.643332044 0.528029115

C -2.120747791 -1.501506149 -0.500911781

C -2.872278315 1.848909447 -0.425961976

C -1.936712403 2.505658439 0.595769095

C -0.606750460 2.837753694 -0.068824706

C 2.337852163 1.205472693 -0.419586082

C 2.305781331 -2.135001042 0.669724506

C -3.552263799 -3.807137397 -0.987728687

C 4.170295178 3.099583734 -1.186114523

C 0.682357146 -4.109512633 0.662000713

C -0.240347983 -4.583598184 1.608485627

C -0.472557381 -5.950041195 1.748841049

C 0.199158892 -6.866109226 0.933373442

C 1.105483202 -6.403386577 -0.020610073

C 1.347789473 -5.034621860 -0.152478362

C -3.941198315 2.721775478 -1.025739551

C -0.349615458 4.231739566 -0.511016197

C -0.652795834 5.310760111 0.335141754

C -0.452609992 6.622806262 -0.095318748

C 0.020141533 6.876423853 -1.383281176

C 0.307740637 5.808382682 -2.238552031

C 0.137072099 4.496665884 -1.803975658

C 3.415151554 -3.004740686 1.169586660

C 3.281244213 -3.791566239 2.324729600

C 4.352746451 -4.544039910 2.805768442

C 5.582116445 -4.524679226 2.144558387

C 5.731147825 -3.737911314 0.999882788

C 4.660888988 -2.984696455 0.520011633

Ni 0.000000000 0.000000000 0.000000000

H -2.315461728 7.515237199 5.779983573

H -1.833837990 8.218047685 4.224921449

H -0.637598676 8.098204885 5.551919690

H -8.636995681 -0.784119861 1.755994314

H -7.151950541 -0.769924561 2.758187555

H -7.032278740 -0.497464812 1.008005258

H -4.386403708 8.262089003 2.153099743

H -4.495810452 10.604202172 2.954338178

H -5.866227536 11.175587189 4.953882423

H -7.131836734 9.382047329 6.128719371

H -7.042992589 7.050945991 5.295675253

H 1.175149465 1.664287363 2.328041052

H 0.465765404 0.884840004 3.737539080

H 1.087561317 2.548614282 3.879061025

H -1.231809652 -0.833514486 2.789920307

H -1.037425732 -2.439711663 4.681987760

H -2.597577949 -2.269519038 6.615670369

H -4.315215119 -0.467927815 6.655738870

H -4.461911805 1.152462525 4.788840008

H -7.370225614 7.860553803 1.532677505

H -9.535470867 8.992914255 1.231830661

H -11.626967216 7.964250232 2.114454969

H -11.505179626 5.768423954 3.289726814

H -9.320959558 4.635779881 3.581608861

H -2.404498522 3.471277021 0.815187096

H -2.746524532 -4.151766306 -1.637919997

H -3.356411602 -4.143544632 0.031429187

H -4.510064220 -4.207216090 -1.334275576

H 5.234357558 3.317372663 -1.315293988

H 3.642286395 3.247761292 -2.129672746

H 3.740821445 3.756458752 -0.428313545

H -0.780594575 -3.873662742 2.227373794

H -1.183895880 -6.300731898 2.492483493

H 0.014270494 -7.932105020 1.041300690

H 1.630099075 -7.106396046 -0.662874023

H 2.062125008 -4.679747415 -0.889391574

H -4.570737548 3.141901208 -0.233486799

H -3.493252473 3.564469584 -1.571422065

H -4.558466095 2.141241813 -1.715141239

H -1.023505662 5.126352420 1.336738138

H -0.674269454 7.443033964 0.581602117

H 0.161871457 7.899836429 -1.722073654

H 0.668803021 5.997892656 -3.246370642

H 0.371943811 3.668881760 -2.463297110

H 2.334211962 -3.810894213 2.853961357

H 4.225658513 -5.142116158 3.704846602

H 6.416056705 -5.113314352 2.519086482

H 6.684386509 -3.710407085 0.477116604

H 4.779248493 -2.363702202 -0.362195855

**Table S4**. Cartesian coordinates of optimized geometry **a8**.

opt b3lyp/6-31g* freq=OK charge=-1 multiplicity =1

127

Ni -4.581250746 2.722964384 3.169379019

S -1.558781829 5.176786610 5.267072927

S -8.030418721 0.145665969 2.317428059

N -5.105615230 4.346661950 3.882859888

N -4.131345675 5.060615099 4.549386396

N -2.815567242 3.299290191 3.751691097

N -1.511387558 2.854128181 3.845966795

N -4.236121558 1.038897590 2.429338683

N -5.322295962 0.192378807 2.340627610

N -6.312041879 2.190554063 2.933933447

N -7.443682423 2.844156472 3.037934136

C -6.341090132 4.853238414 3.932121989

C -2.973707999 4.453254661 4.456047176

C -1.037858201 1.873050430 3.145933828

C -1.929526867 1.225351664 2.125242774

C -2.993366934 0.536717086 2.697117792

C -6.415355490 0.855866464 2.537964521

C -7.473861772 4.109414125 3.443813599

C -2.355707242 6.643529695 6.020326822

C -7.515828589 -1.540887915 1.832618678

C -6.551409494 6.171986712 4.610593936

C -5.975606360 7.347186157 4.102451269

C -6.191669860 8.573862898 4.726928126

C -6.976226554 8.647159651 5.882171522

C -7.542710109 7.483574325 6.402573177

C -7.333932689 6.256578862 5.769151045

C 0.383228409 1.471888924 3.434027623

C -2.787691653 -0.553632064 3.678535053

C -1.711012601 -1.444442854 3.526926776

C -1.486994719 -2.461824931 4.455477752

C -2.349416486 -2.621514498 5.541847280

C -3.436071099 -1.753796122 5.692923880

C -3.655241095 -0.732570651 4.771608795

C -8.847950209 4.695126750 3.389362172

C -9.101728211 5.980626669 2.881854803

C -10.403006684 6.476161725 2.794793501

C -11.485728164 5.696114143 3.204505830

C -11.250311069 4.409765377 3.696709175

C -9.950052243 3.916120480 3.785971616

S -3.468246091 -2.290416641 -1.310891472

S 3.871377618 1.933167499 -0.468888165

N 0.182731693 -1.843301151 -0.075411329

N -0.922824729 -2.529157059 -0.491834490

N -1.780615544 -0.372278486 -0.425740214

N -2.877746652 0.383300079 -0.759539251

N -0.000000000 1.896273926 0.000000000

N 1.243537673 2.458760151 -0.333284918

N 1.808218544 0.231313203 -0.000000000

N 2.809534529 -0.610572656 0.219972971

C 1.281315286 -2.547660504 0.178291559

C -1.923013249 -1.680823907 -0.692716074

C -3.088717612 1.508976351 -0.180848980

C -2.226402969 2.141192700 0.917901737

C -0.959983001 2.722633532 0.302371094

C 2.160350415 1.536490373 -0.258559560

C 2.566830236 -1.893785028 0.385160637

C -3.060971347 -4.066811489 -1.476475512

C 3.726995179 3.731015397 -0.771154910

C 1.204798814 -4.040556601 0.119634769

C 0.364877861 -4.746474206 0.996098506

C 0.308773256 -6.137910505 0.956796442

C 1.076790043 -6.847919906 0.028675939

C 1.901767803 -6.154283981 -0.856706155

C 1.967736236 -4.760382731 -0.808686436

C -4.268745364 2.309433988 -0.661494681

C -0.887621117 4.183419565 0.046019268

C -1.310072166 5.096467491 1.025848616

C -1.283696497 6.467926342 0.770426104

C -0.868015518 6.944172724 -0.473383291

C -0.462552043 6.041125565 -1.460813604

C -0.459622227 4.673202853 -1.201267460

C 3.784932187 -2.673092084 0.767154961

C 3.770234336 -3.613505124 1.809646268

C 4.935710112 -4.279856919 2.188293254

C 6.141750084 -4.019168353 1.535274713

C 6.171630436 -3.078248889 0.503153569

C 5.007314117 -2.411161685 0.125761068

Ni 0.000000000 0.000000000 0.000000000

H -3.146397799 6.348082860 6.711958594

H -2.782852155 7.301801818 5.262001033

H -1.559215243 7.163832604 6.561982698

H -8.439438160 -2.094538604 1.638549953

H -6.951574717 -2.021969359 2.634013635

H -6.896149566 -1.512117936 0.934403884

H -5.354658997 7.289609555 3.213675504

H -5.744547272 9.475216667 4.313928041

H -7.142633244 9.604211124 6.370968890

H -8.152911773 7.527621624 7.301510168

H -7.785332272 5.354354809 6.171162480

H 0.994858367 1.484228670 2.525455586

H 0.412622297 0.445099819 3.821087097

H 0.822430481 2.141857207 4.178683360

H -1.070761333 -1.337413638 2.657169859

H -0.644530583 -3.137420990 4.323265478

H -2.181438471 -3.418511184 6.262613654

H -4.111172433 -1.869927969 6.537624070

H -4.491469863 -0.053172171 4.898335088

H -8.274129581 6.596487595 2.546127080

H -10.569312168 7.474301401 2.395741449

H -12.499867800 6.082965080 3.136464416

H -12.083348183 3.786747867 4.014898867

H -9.769457038 2.912224166 4.156359442

H -2.808249035 3.003021682 1.261548824

H -2.229164461 -4.219259680 -2.166106169

H -2.807492364 -4.504814659 -0.509980654

H -3.966441691 -4.536899358 -1.872304017

H 4.752917539 4.096946045 -0.870785092

H 3.168977029 3.931968973 -1.687280113

H 3.230914357 4.224337098 0.065942645

H -0.250057564 -4.197032468 1.702402603

H -0.340374357 -6.669382027 1.648270167

H 1.029289549 -7.933684599 -0.003538612

H 2.499940176 -6.695869773 -1.585281307

H 2.619441848 -4.225288258 -1.492942172

H -4.932919970 2.540082218 0.178840480

H -3.939476122 3.265686231 -1.092449035

H -4.819415579 1.749507140 -1.420808698

H -1.637988566 4.738328277 1.994783303

H -1.595533313 7.158739413 1.548669870

H -0.861793361 8.012490760 -0.675617992

H -0.144950098 6.403864363 -2.435250876

H -0.133437690 3.974355249 -1.963049402

H 2.842058743 -3.821239056 2.331821131

H 4.899788024 -5.000613172 3.001563262

H 7.049207779 -4.540744160 1.829720246

H 7.105040846 -2.862524290 -0.011543290

H 5.031974756 -1.671160393 -0.667789702

**Table S5**. Cartesian coordinates of optimized geometry **a7**.

opt b3lyp/6-31g* freq=OK charge=-1 multiplicity =1

127

Ni -1.948538560 5.330256419 2.710379858

S 2.137142888 3.856635752 3.594456677

S -5.930553371 7.173125193 2.848741746

N -0.606280485 6.562974975 3.023201365

N 0.646310984 6.049615740 3.275003629

N -0.482305813 4.036490582 2.890390458

N -0.277045237 2.682717310 3.087660959

N -3.410600909 4.242745254 2.299524870

N -4.655530056 4.802996916 2.503212806

N -3.252414695 6.605392522 2.827737421

N -3.183039095 7.911532053 2.936936147

C -0.727438583 7.891222348 3.125634604

C 0.633915438 4.737480323 3.221253481

C -1.092239962 1.776854512 2.649648538

C -2.248272571 2.242132319 1.830723298

C -3.198045633 2.918685476 2.585836286

C -4.535898412 6.075743401 2.711490800

C -2.013495439 8.534518827 3.032369923

C 3.256108247 5.270438481 3.911082460

C -7.263875249 5.931790644 2.696411300

C 0.493920579 8.701268857 3.436160419

C 1.568207396 8.765562976 2.534409824

C 2.691355918 9.539333787 2.819327737

C 2.769325898 10.252249532 4.019853162

C 1.713635140 10.183962833 4.928916186

C 0.583965075 9.416318490 4.637080926

C -0.840171419 0.365456379 3.107941983

C -3.799503722 2.305944593 3.798478917

C -4.182269493 0.954245325 3.786297330

C -4.748985071 0.360251770 4.915464775

C -4.956577507 1.113090621 6.072463063

C -4.593524571 2.464148294 6.092193046

C -4.026281242 3.056296392 4.966817100

C -2.165152254 10.022180927 3.064863027

C -1.360743669 10.884740790 2.301020972

C -1.570298111 12.263988101 2.313138825

C -2.595046442 12.817074145 3.083057848

C -3.412782619 11.970994022 3.836309028

C -3.201406345 10.593358781 3.825479724

S -3.793999704 -1.994636118 -0.825906320

S 4.085686800 1.432357364 -0.470857661

N -0.043869529 -1.847061773 0.168137316

N -1.262269951 -2.431785920 -0.071916699

N -1.843567631 -0.187102545 -0.312270160

N -2.863182676 0.592138916 -0.801289036

N 0.214521666 1.871515743 -0.199394246

N 1.534495684 2.243275515 -0.506487357

N 1.820738299 0.000000000 0.000000000

N 2.711496831 -0.937096111 0.264004934

C 0.967580974 -2.661570057 0.445915664

C -2.151301492 -1.503505510 -0.364124807

C -2.944993858 1.845334045 -0.550013140

C -2.036311711 2.692651613 0.358528772

C -0.645222358 2.853142047 -0.223605292

C 2.335459035 1.240581268 -0.323672728

C 2.330497348 -2.168296672 0.537540831

C -3.624673428 -3.812228553 -0.687281065

C 4.156265268 3.237293278 -0.759689819

C 0.698661188 -4.130024291 0.557237142

C -0.119003274 -4.634186223 1.580248554

C -0.351023857 -6.003516539 1.691583878

C 0.216200937 -6.890470561 0.771536945

C 1.019207044 -6.396490622 -0.256565662

C 1.262391661 -5.025423219 -0.360003033

C -4.119871254 2.577647656 -1.146021434

C -0.333729656 4.121556665 -0.944395079

C -0.522262916 5.378275220 -0.357055482

C -0.303668126 6.543966239 -1.091770358

C 0.089138881 6.470517814 -2.428463488

C 0.270175099 5.219862485 -3.027137150

C 0.070514505 4.055070234 -2.290681172

C 3.467966414 -3.062904530 0.915309080

C 3.415264793 -3.909797185 2.033972752

C 4.515777234 -4.687084550 2.395263481

C 5.694596558 -4.631946187 1.649430274

C 5.764058489 -3.784395556 0.541309024

C 4.664265382 -3.007622600 0.180362447

Ni 0.000000000 0.000000000 0.000000000

H 2.891145744 5.883948650 4.736426632

H 3.364202340 5.901154167 3.026933054

H 4.221733492 4.825727519 4.172318274

H -8.204293369 6.485479872 2.775390089

H -7.197879859 5.188931548 3.493912599

H -7.209836873 5.420404790 1.733296727

H 1.517761766 8.197979022 1.610110397

H 3.510662855 9.582787614 2.105438409

H 3.647376823 10.853634890 4.243249707

H 1.764062613 10.731300235 5.867138983

H -0.240582005 9.374192361 5.342640199

H -0.981364923 -0.346634119 2.291539902

H -1.557317936 0.098538555 3.895783023

H 0.172010340 0.273732359 3.513410513

H -4.044483631 0.385121032 2.871652438

H -5.040511768 -0.687150624 4.885428398

H -5.404184344 0.654649659 6.951407568

H -4.749375025 3.056245797 6.990997892

H -3.737359091 4.102041560 4.986787120

H -0.566025912 10.473436125 1.687817260

H -0.932851358 12.906762435 1.710103290

H -2.757366372 13.892401910 3.092527873

H -4.219217217 12.385092484 4.437585693

H -3.845451094 9.936081570 4.400631481

H -2.465560079 3.691853520 0.298479529

H -2.854318525 -4.187783103 -1.362621244

H -3.378288250 -4.111426223 0.332709841

H -4.600959549 -4.218238156 -0.969537135

H 5.219341963 3.480418012 -0.845205478

H 3.632746328 3.512019695 -1.676599094

H 3.713437246 3.770462316 0.083338080

H -0.573525997 -3.944799216 2.285074427

H -0.979569313 -6.379320907 2.495191550

H 0.031855000 -7.958550547 0.857105416

H 1.463437424 -7.077180951 -0.978482573

H 1.898085774 -4.645669535 -1.154414797

H -4.709599476 3.055426911 -0.353055651

H -3.777282453 3.380905312 -1.813087026

H -4.752696476 1.888309420 -1.710030267

H -0.826682916 5.436567261 0.680739147

H -0.450188646 7.508044283 -0.612216184

H 0.249337147 7.379533230 -3.003094278

H 0.568134226 5.151193050 -4.070512157

H 0.216535195 3.085704260 -2.756160191

H 2.510343278 -3.955737340 2.630679019

H 4.451912047 -5.331673795 3.268573795

H 6.551369507 -5.239112909 1.931387299

H 6.677756813 -3.727304572 -0.045667451

H 4.721825014 -2.339677760 -0.673205571

**Table S6**. Cartesian coordinates of optimized geometry **b1**.

opt b3lyp/6-31g* freq=OK charge=-1 multiplicity =1

127

Ni -2.236399985 4.768501035 4.137713110

S 1.941303455 3.394522361 4.701828338

S -6.362356936 6.159892305 4.721780383

N -1.007899726 5.934124709 4.884535598

N 0.276906508 5.459494522 5.034437233

N -0.670928359 3.607540765 3.975902843

N -0.352794284 2.309549595 3.683140026

N -3.605112914 3.771627971 3.312893019

N -4.893422208 4.130057129 3.678958187

N -3.647074537 5.830848533 4.608798155

N -3.689430589 7.042325636 5.113618233

C -1.244052737 7.149735122 5.388190786

C 0.378806737 4.240020145 4.561356918

C -1.082619551 1.502906779 2.990065785

C -2.313562607 2.006095959 2.314587722

C -3.239874017 2.453570724 3.265829658

C -4.881561403 5.271173076 4.287267841

C -2.579221118 7.692040228 5.442171238

C 2.927180768 4.700971527 5.522448282

C -7.588559287 4.934086099 4.142446208

C -0.105514400 7.908959436 5.997785694

C 0.985134448 8.328301886 5.218968864

C 2.030137769 9.052408378 5.789130736

C 2.012678853 9.358271761 7.154023757

C 0.940418633 8.934603022 7.939622536

C -0.111393513 8.218174217 7.364347629

C -0.637408610 0.065362737 3.019358768

C -3.636603813 1.570870150 4.393452457

C -3.878885008 0.205082283 4.168335515

C -4.254662589 -0.637587751 5.216067881

C -4.411330044 -0.124704439 6.505412757

C -4.189323805 1.236759343 6.739258577

C -3.810144829 2.076408463 5.695029623

C -2.855302207 9.083537624 5.917269444

C -2.109641996 10.194265146 5.486376122

C -2.433080546 11.485457688 5.905883699

C -3.515694257 11.701450337 6.760995714

C -4.275682414 10.608543997 7.186172339

C -3.950701857 9.318619357 6.768482547

S -3.651751774 -2.312643674 -0.608786762

S 3.854457588 1.912582249 -0.666268466

N 0.159637938 -1.849128334 -0.034659660

N -1.014300493 -2.537581295 -0.206408758

N -1.839801809 -0.358790493 -0.124675952

N -2.994120227 0.337811107 -0.377162789

N -0.000000000 1.897863048 -0.000000000

N 1.247421589 2.440542001 -0.360142670

N 1.801513890 0.198105467 -0.175858864

N 2.805009283 -0.657956445 -0.115530928

C 1.271110240 -2.572939521 0.037700624

C -2.020323800 -1.690292218 -0.288153059

C -3.177086244 1.549504816 -0.004624744

C -2.265159116 2.464741052 0.832448665

C -0.937342694 2.790253202 0.172642900

C 2.155227451 1.516020781 -0.383039198

C 2.580728586 -1.946091757 0.044335180

C -3.286458244 -4.104851321 -0.701174772

C 3.716760288 3.733888022 -0.774871803

C 1.161122491 -4.065899844 0.005590530

C 0.525643486 -4.761416264 1.045987480

C 0.443800841 -6.152414684 1.021605588

C 0.980483368 -6.870423753 -0.051807649

C 1.602029794 -6.185987019 -1.096680169

C 1.695581952 -4.792857221 -1.065638590

C -4.514320964 2.155691581 -0.353650561

C -0.768688632 4.184475456 -0.331416274

C -1.033399873 5.288570558 0.489140315

C -0.947525604 6.585091444 -0.019179222

C -0.621908348 6.794886039 -1.359829534

C -0.373235648 5.697508401 -2.190918202

C -0.434575490 4.403113572 -1.680472380

C 3.836331208 -2.745775052 0.190942764

C 3.998897303 -3.706912069 1.201840635

C 5.204924080 -4.392244273 1.349856703

C 6.276117421 -4.129163730 0.493844190

C 6.131160685 -3.166705103 -0.508410310

C 4.925914449 -2.481713870 -0.656192513

Ni 0.000000000 0.000000000 0.000000000

H 2.488758732 4.981947597 6.481826680

H 3.007057052 5.593128443 4.898293857

H 3.919276501 4.264075108 5.676535447

H -8.571910031 5.364038714 4.356778483

H -7.471056575 3.985118967 4.670546938

H -7.482106436 4.756605715 3.070029792

H 1.009137117 8.076798794 4.162706231

H 2.863219335 9.375090865 5.168504379

H 2.829969183 9.920729154 7.599311178

H 0.917032128 9.163713426 9.002698131

H -0.949595836 7.899164934 7.977206772

H -0.674170738 -0.382779880 2.023764625

H -1.314105046 -0.518630394 3.658254920

H 0.376692425 -0.009230791 3.423122794

H -3.788577989 -0.178598998 3.156068748

H -4.438834327 -1.691777320 5.021363093

H -4.710961081 -0.777825803 7.322269407

H -4.306888093 1.643260964 7.741311391

H -3.625629779 3.130081299 5.877418915

H -1.271339186 10.048048694 4.813033384

H -1.838919702 12.326309767 5.554358939

H -3.766802964 12.708181071 7.088176053

H -5.125750873 10.759889563 7.848381138

H -4.549560162 8.471995990 7.088883460

H -2.797917691 3.415457677 0.833532860

H -2.576290463 -4.320993034 -1.501129700

H -2.882734878 -4.477382109 0.241828263

H -4.246056205 -4.586903911 -0.912099845

H 4.736461699 4.096565838 -0.933555026

H 3.078039790 4.033601640 -1.607193739

H 3.312879236 4.141414723 0.153492219

H 0.093977678 -4.203793195 1.871746537

H -0.043719834 -6.677349842 1.839689601

H 0.913213600 -7.955620538 -0.071648940

H 2.020886074 -6.734415532 -1.936966203

H 2.191137211 -4.264245429 -1.874893608

H -5.017074428 2.517258213 0.552682263

H -4.383818520 3.027206593 -1.010863158

H -5.145957000 1.419433206 -0.856296977

H -1.299664301 5.127192712 1.528052961

H -1.147163095 7.426824200 0.638678494

H -0.567610363 7.805206330 -1.758533724

H -0.129384023 5.850417461 -3.239696224

H -0.233016115 3.553676807 -2.325247542

H 3.179798065 -3.914881503 1.882377538

H 5.308070384 -5.128686276 2.143423155

H 7.215288461 -4.664868377 0.609521874

H 6.959024111 -2.947771260 -1.178919969

H 4.816615555 -1.725071822 -1.427001894

**Table S7**. Cartesian coordinates of optimized geometry **b2**.

opt b3lyp/6-31g* freq=OK charge=-1 multiplicity =1

127

Ni -2.541647243 5.691956686 4.210661338

S -0.985795650 2.372763269 6.599381324

S -5.115175639 9.012724790 2.811512010

N -2.002115037 6.058086041 5.951551836

N -1.587035021 4.984403726 6.683878285

N -1.882176644 3.971385228 4.610535851

N -1.628735889 2.790756716 4.001648437

N -3.137551229 5.683637827 2.274241471

N -4.179177906 6.591571567 2.066354030

N -3.389598195 7.332513609 4.107293889

N -3.309258679 8.408498302 4.870514470

C -2.000954055 7.234439907 6.579956189

C -1.526506970 3.919239620 5.912432847

C -1.826547552 2.552167057 2.736251923

C -2.277469223 3.503696275 1.727445275

C -3.341478183 4.372557886 2.218764554

C -4.160005282 7.518694591 2.962393403

C -2.623198537 8.399356575 5.996899686

C -0.760892617 2.879786842 8.346645334

C -5.930805270 8.652863206 1.214569530

C -1.434000057 7.300761722 7.963881374

C -0.075625937 7.033376139 8.197815849

C 0.454034717 7.117278273 9.483846477

C -0.368649974 7.454440526 10.563977463

C -1.722320510 7.708415497 10.343904864

C -2.249759512 7.635012155 9.052624404

C -1.519731497 1.100774713 2.433277551

C -4.579315031 3.777497265 2.812838058

C -4.950575271 2.449171023 2.529128337

C -6.096090713 1.880806491 3.083256182

C -6.911038245 2.629139195 3.934539341

C -6.553602078 3.944767081 4.240912388

C -5.401592834 4.506828268 3.697956512

C -2.567584470 9.743245924 6.651762200

C -1.376070249 10.284470155 7.162338602

C -1.347917790 11.566668804 7.712483514

C -2.508586309 12.341189715 7.760902078

C -3.697959256 11.820274537 7.245139353

C -3.725969802 10.539067222 6.695858164

S -3.856736061 -1.961031732 -0.613362172

S 4.107419504 1.470334825 -0.182553439

N -0.026160780 -1.861005119 0.015474778

N -1.255359479 -2.433452691 -0.191840678

N -1.857012690 -0.184888000 -0.171138081

N -2.953040320 0.590598165 -0.376883247

N 0.210417295 1.913278689 -0.049267216

N 1.561418690 2.295028328 -0.169362223

N 1.824937134 0.000000000 0.000000000

N 2.721725723 -0.957599636 0.135297523

C 0.996252617 -2.700334126 0.149233687

C -2.170743619 -1.498277443 -0.300936719

C -3.042056791 1.855077112 -0.190262897

C -2.076919793 2.936614404 0.299399426

C -0.596028658 2.949962002 -0.033385587

C 2.348423212 1.272781274 -0.116431342

C 2.357422509 -2.219005464 0.250602575

C -3.663649175 -3.781316719 -0.687860188

C 4.187562277 3.293224887 -0.323844457

C 0.730187865 -4.173761124 0.105362905

C -0.023620090 -4.800739535 1.109834621

C -0.246591603 -6.176038165 1.074022670

C 0.265725541 -6.945201225 0.024218283

C 1.005112877 -6.327934439 -0.985135062

C 1.239597361 -4.951568181 -0.941913442

C -4.362237947 2.439698299 -0.663386197

C -0.067924035 4.304753022 -0.411047686

C -0.010736908 5.382334578 0.481325592

C 0.404475542 6.641446464 0.035803363

C 0.760076901 6.840647735 -1.297235526

C 0.704492597 5.767977982 -2.193991230

C 0.299368844 4.510423630 -1.753614918

C 3.512937521 -3.143391987 0.469958652

C 3.510056313 -4.127696663 1.471840775

C 4.629283840 -4.931744207 1.688140665

C 5.777984024 -4.766814536 0.911677340

C 5.798422049 -3.782861496 -0.079938209

C 4.679622772 -2.979655477 -0.296277277

Ni 0.000000000 0.000000000 0.000000000

H -1.699150928 3.228275905 8.782833158

H -0.014138353 3.670130131 8.440935745

H -0.419853534 1.980105725 8.869363145

H -6.556092134 9.523234312 0.992992179

H -6.549311400 7.755385137 1.283522220

H -5.189383225 8.512926392 0.425274461

H 0.560613735 6.754755960 7.362859080

H 1.510304529 6.915164709 9.645207442

H 0.044490094 7.516546986 11.568072994

H -2.372300791 7.968079995 11.176397857

H -3.302299461 7.843074359 8.884379160

H -0.876343379 0.972504523 1.565384470

H -2.442286310 0.546413547 2.213584080

H -1.037817977 0.644663081 3.301168295

H -4.334267940 1.855564271 1.865326678

H -6.350902353 0.851305413 2.844413631

H -7.809216071 2.190148754 4.362724205

H -7.165464572 4.533264885 4.920557221

H -5.121741926 5.510316069 3.986301197

H -0.462504827 9.700347771 7.124329810

H -0.411196691 11.962617330 8.098072083

H -2.485932897 13.339918406 8.191385644

H -4.609586941 12.413302208 7.271197998

H -4.646959430 10.139480898 6.283589950

H -2.415739991 3.787537331 -0.312145998

H -2.978271830 -4.072891467 -1.485652584

H -3.296425760 -4.181394280 0.258782659

H -4.665057289 -4.172005664 -0.894014308

H 5.252587274 3.538360607 -0.372502550

H 3.679800099 3.641011877 -1.225008138

H 3.732399869 3.768534668 0.546753221

H -0.435913297 -4.201938868 1.916466069

H -0.825167316 -6.648448617 1.864450808

H 0.088309312 -8.017782553 -0.004707701

H 1.406626202 -6.916275189 -1.806743647

H 1.826371865 -4.476162218 -1.722838016

H -4.737127292 3.218512180 0.006024167

H -4.220800185 2.904637644 -1.650838414

H -5.111967046 1.651133538 -0.760186966

H -0.301953881 5.237452979 1.513785960

H 0.438137657 7.467567566 0.741428992

H 1.076455369 7.823491349 -1.639194072

H 0.975698982 5.911343878 -3.237652759

H 0.263353835 3.676705499 -2.450052753

H 2.629733873 -4.261179440 2.091822950

H 4.603910020 -5.684227542 2.473028979

H 6.649296228 -5.394988002 1.080592336

H 6.688210704 -3.639498349 -0.688783611

H 4.699192053 -2.207010247 -1.058721407

**Table S8**. Cartesian coordinates of optimized geometry **b3**.

opt b3lyp/6-31g* freq=OK charge=-1 multiplicity =1

127

Ni -2.530540769 5.422922288 4.901009496

S 0.963268216 2.990648922 5.784054415

S -6.225305307 7.677845027 4.544701080

N -1.597157668 5.846425882 6.432133127

N -0.454658720 5.114280883 6.632433294

N -1.180926440 4.184459403 4.633179709

N -1.186349360 2.998898831 3.983179353

N -3.404820443 5.256098614 3.244515526

N -4.565700789 6.012743095 3.203763865

N -3.881309922 6.552316253 5.336706292

N -4.087104689 7.339260381 6.373495345

C -1.933132476 6.721221101 7.392523296

C -0.326580575 4.212527494 5.681597182

C -1.683092618 2.804413162 2.802230565

C -2.227383426 3.701967041 1.764413274

C -3.017493428 4.839762740 1.980892854

C -4.792315530 6.647359173 4.311374695

C -3.172693340 7.444724054 7.333372390

C 1.812636055 3.568260912 7.298721130

C -7.061420436 7.374143896 2.947949717

C -1.025564935 6.859729318 8.574787754

C 0.275863333 7.368120383 8.433308465

C 1.109988968 7.510657577 9.540484636

C 0.664419070 7.133457235 10.811686041

C -0.621392660 6.613596361 10.961862986

C -1.458837612 6.479118334 9.852080774

C -1.670749351 1.310666926 2.509226026

C -3.533265063 5.704273962 0.839574929

C -4.855008409 5.653117765 0.366463843

C -5.293958060 6.514101181 -0.638648498

C -4.427404644 7.468207739 -1.180335448

C -3.124628102 7.555684283 -0.694506678

C -2.685232456 6.686149601 0.308972618

C -3.572467544 8.433785624 8.382747812

C -2.703175060 9.424978933 8.869609274

C -3.135693516 10.364857443 9.806119086

C -4.450659177 10.343626022 10.275154156

C -5.330979531 9.373587488 9.789132734

C -4.897473014 8.433710003 8.854762977

S -3.853981226 -1.911880549 -0.699946942

S 4.088404584 1.481045989 -0.247153367

N -0.018999964 -1.855975072 -0.101628608

N -1.240740752 -2.408817141 -0.383793357

N -1.842493476 -0.168359030 -0.189204480

N -2.963674987 0.588330034 -0.123525930

N 0.192344847 1.910307958 -0.020139252

N 1.540653506 2.290819300 -0.183835242

N 1.824328359 -0.000000000 -0.000000000

N 2.725440090 -0.958666921 0.111573791

C 1.001789034 -2.700392813 0.008136910

C -2.161099147 -1.471409508 -0.403101864

C -3.075722578 1.837035730 0.142404971

C -2.090557896 2.995380405 0.373257205

C -0.600640142 2.948703602 0.049741000

C 2.335112952 1.270983973 -0.135592476

C 2.359247074 -2.222665337 0.172455209

C -3.653372894 -3.710158537 -0.988955805

C 4.157545955 3.302935012 -0.404076152

C 0.743230607 -4.168470355 -0.135153425

C -0.044403821 -4.855674092 0.801722920

C -0.261026664 -6.226612666 0.675103305

C 0.291811845 -6.930235252 -0.399758679

C 1.065443656 -6.252436872 -1.342398719

C 1.293538057 -4.880942281 -1.207930646

C -4.528268524 2.269554600 0.180760543

C 0.016709499 4.307547230 -0.169356858

C 0.552443804 5.055482961 0.888196950

C 1.132890388 6.300215235 0.638321325

C 1.184878915 6.809105979 -0.663330718

C 0.650728766 6.065603458 -1.717320778

C 0.068695756 4.819776738 -1.470869455

C 3.506890320 -3.157641060 0.386740227

C 3.469635245 -4.182584151 1.346026880

C 4.579547075 -4.999176637 1.563254598

C 5.751906440 -4.806321901 0.829902026

C 5.805784103 -3.782252964 -0.118999676

C 4.696615572 -2.966042686 -0.335993677

Ni 0.000000000 0.000000000 0.000000000

H 1.128090031 3.578335930 8.148589242

H 2.223702898 4.571278067 7.166422396

H 2.622815329 2.853835140 7.475449635

H -7.970695683 7.983036753 2.965433109

H -7.324305953 6.319213668 2.838594404

H -6.427515767 7.671370073 2.110463614

H 0.629354820 7.646572595 7.444928964

H 2.112210914 7.913463053 9.411699687

H 1.316366662 7.242610598 11.675398315

H -0.978191956 6.313036144 11.944504909

H -2.462189044 6.081691648 9.974456957

H -1.126546338 1.036198092 1.603751095

H -2.691309477 0.923683456 2.397270327

H -1.199231508 0.808094892 3.356097511

H -5.551585876 4.954099654 0.811569538

H -6.321671459 6.448839746 -0.988759026

H -4.771289811 8.141739238 -1.962110814

H -2.439436212 8.301898869 -1.088791203

H -1.675860053 6.779754559 0.691366516

H -1.681304199 9.465584734 8.508024346

H -2.441198104 11.122367830 10.163249947

H -4.786391322 11.076266401 11.005977206

H -6.360581898 9.346579038 10.139661715

H -5.585164902 7.688708509 8.467280644

H -2.454487753 3.749289530 -0.337010356

H -3.004316544 -3.903240054 -1.845154373

H -3.241149555 -4.210193269 -0.111035121

H -4.660930293 -4.085611596 -1.193096109

H 5.220613435 3.551064756 -0.474289148

H 3.633072464 3.639909029 -1.299802608

H 3.716774082 3.784449655 0.470261668

H -0.487146494 -4.307451607 1.627948570

H -0.865776831 -6.746698046 1.414262261

H 0.119358383 -7.999309099 -0.499635261

H 1.498663575 -6.789729742 -2.182576973

H 1.906878260 -4.358803551 -1.936821446

H -4.780983434 2.719937672 1.146531700

H -4.743865238 3.014730621 -0.592974994

H -5.159152251 1.393777214 0.018273278

H 0.513499503 4.666980095 1.900401613

H 1.538854286 6.874543805 1.467092933

H 1.635125897 7.780779927 -0.852321063

H 0.682488193 6.453119079 -2.732939931

H -0.342249201 4.240792368 -2.294472708

H 2.569739109 -4.338193988 1.931926098

H 4.528112567 -5.783571779 2.314834123

H 6.615948354 -5.444242049 0.999300408

H 6.714352632 -3.617554708 -0.693611363

H 4.741660721 -2.162607481 -1.064814595

**Table S9**. Cartesian coordinates of optimized geometry **c1**.

opt b3lyp/6-31g* freq=OK charge=-1 multiplicity =1

127

Ni -1.512718368 6.538877977 3.689795899

S 1.495270375 3.763614335 5.159142656

S -4.918683640 9.216031978 3.332938560

N -0.156333434 7.256581282 4.705855896

N 0.798379711 6.359398766 5.109674464

N -0.547277455 4.956837011 3.832609495

N -0.847079433 3.636807754 3.670448642

N -2.903182043 5.920640258 2.603695403

N -3.959707257 6.817113906 2.532382934

N -2.515511559 8.042086086 3.849051480

N -2.270663592 9.219337080 4.383205227

C -0.041128891 8.514006620 5.156525365

C 0.504236134 5.155753141 4.663823704

C -1.570805545 3.159926546 2.703660380

C -2.199449871 3.820699624 1.544199281

C -2.833572584 5.066760485 1.532403336

C -3.732082126 7.895098138 3.214345310

C -1.095541225 9.470193681 4.954610378

C 2.730432706 4.625027603 6.199063351

C -6.284949587 8.457004794 2.385284735

C 1.159144440 8.867447423 5.977293867

C 2.450739035 8.815561769 5.428568568

C 3.564474847 9.160881648 6.191181653

C 3.410801170 9.551364093 7.525154450

C 2.133898476 9.592627520 8.085219041

C 1.018117483 9.254384772 7.316595097

C -1.718781887 1.650153336 2.819347293

C -3.527924107 5.598337245 0.290789228

C -4.913310698 5.474286002 0.102168981

C -5.540873027 6.036753588 -1.008688279

C -4.796558955 6.750922731 -1.952295278

C -3.424797435 6.907655998 -1.761140211

C -2.798818755 6.345367096 -0.644663286

C -0.975404722 10.893504718 5.396586562

C 0.170302189 11.669582213 5.152330815

C 0.224296905 13.011764455 5.529267021

C -0.868668397 13.615993767 6.153185309

C -2.020517374 12.861200741 6.389065938

C -2.073132122 11.519798478 6.014047732

S -3.860146162 -1.936044813 -0.590334671

S 4.089170370 1.474850178 -0.277592008

N -0.017772300 -1.857555020 -0.044786509

N -1.242296082 -2.420042612 -0.293435592

N -1.844599235 -0.175078771 -0.165805738

N -2.969779645 0.580515520 -0.110856752

N 0.192927795 1.905767278 -0.084933673

N 1.539878526 2.282168894 -0.257464490

N 1.824198549 0.000000000 0.000000000

N 2.725899663 -0.954635561 0.133128335

C 1.004049743 -2.698537180 0.083066321

C -2.163751109 -1.484618598 -0.331162835

C -3.077671165 1.841978395 0.085055256

C -2.087110684 3.011485486 0.212565777

C -0.606525187 2.938938959 -0.130474611

C 2.335977719 1.266424098 -0.171874261

C 2.361589001 -2.216957841 0.226839380

C -3.660535557 -3.742315870 -0.821805934

C 4.157177860 3.292335088 -0.474892627

C 0.744752642 -4.169828236 -0.015746699

C -0.032953639 -4.831142430 0.947136659

C -0.249227078 -6.204854938 0.860861946

C 0.294018763 -6.937592181 -0.198921205

C 1.057749108 -6.285879122 -1.167160133

C 1.285470158 -4.911360538 -1.073136334

C -4.528756520 2.276462425 0.151998665

C -0.015424570 4.264221864 -0.538528397

C 0.519301298 5.166755170 0.389047907

C 1.048683460 6.382570663 -0.046558647

C 1.052898044 6.707399742 -1.406160148

C 0.523901550 5.807031314 -2.332633329

C -0.008621223 4.590497086 -1.900380248

C 3.511700256 -3.144748702 0.458139487

C 3.481988739 -4.145288277 1.442575416

C 4.594338401 -4.954778063 1.673160242

C 5.761926286 -4.778915540 0.928365559

C 5.808518742 -3.778833469 -0.045570790

C 4.696862147 -2.969826341 -0.275967737

Ni 0.000000000 0.000000000 0.000000000

H 2.247956103 5.146655625 7.027237205

H 3.306692443 5.346741274 5.617325607

H 3.391958127 3.841649142 6.581913249

H -7.095725014 9.192042012 2.386467303

H -6.620656057 7.531943903 2.859006084

H -5.976792860 8.240029060 1.361050098

H 2.574077073 8.497383328 4.397885632

H 4.555907399 9.121757853 5.745647391

H 4.280327572 9.818384094 8.121192493

H 2.001667734 9.890701617 9.122610634

H 0.025108004 9.295368160 7.754182572

H -1.229983205 1.112201566 2.000150921

H -2.772570527 1.346537429 2.822868056

H -1.253694738 1.333278453 3.755177144

H -5.504809973 4.954131422 0.847023088

H -6.615602297 5.924646414 -1.132482134

H -5.285516511 7.188899229 -2.819297461

H -2.832228321 7.474551504 -2.474909880

H -1.736981909 6.498619210 -0.493072522

H 1.025185959 11.220583808 4.657983135

H 1.123855924 13.588420600 5.325489077

H -0.825513414 14.662148521 6.447402805

H -2.883091601 13.318144616 6.869318666

H -2.972713352 10.937622624 6.185551010

H -2.468804846 3.708520941 -0.544112001

H -3.023173623 -3.963080246 -1.679828607

H -3.234991995 -4.212320708 0.066022575

H -4.670131865 -4.125568171 -0.999199398

H 5.220177237 3.539556676 -0.547929549

H 3.634244177 3.608898650 -1.378651942

H 3.714565624 3.791334600 0.388351467

H -0.468296142 -4.260556877 1.761713713

H -0.846248388 -6.704500284 1.619770784

H 0.121850252 -8.008879120 -0.267294041

H 1.483542352 -6.845869877 -1.995895502

H 1.891163888 -4.409326060 -1.821885124

H -4.750569869 2.747233303 1.115399130

H -4.769434610 3.008043397 -0.626455703

H -5.164137346 1.397813358 0.028526692

H 0.518447156 4.923367495 1.445606574

H 1.452023190 7.078308602 0.684290073

H 1.462075228 7.657844701 -1.739340304

H 0.520617286 6.049421989 -3.392485686

H -0.418495626 3.890369321 -2.624195281

H 2.586032265 -4.287341537 2.037576177

H 4.548617476 -5.720107531 2.444092977

H 6.627913861 -5.411234725 1.108247502

H 6.713371394 -3.627235580 -0.629307703

H 4.736445923 -2.184733162 -1.024365379

**Table S10**. Cartesian coordinates of optimized geometry **d1**.

opt b3lyp/6-31g* freq=OK charge=-1 multiplicity =1

127

Ni -4.386852301 7.477328089 -0.263689271

S -3.300009856 7.276250680 3.948656145

S -5.716171861 7.981700188 -4.377895133

N -5.039453539 8.817279037 0.829612372

N -4.737732614 8.671692743 2.154348832

N -3.589186848 6.842915526 1.289867908

N -2.631568396 5.961194652 1.657417748

N -3.897537673 6.090664236 -1.447501047

N -4.365982513 6.324590892 -2.730822976

N -5.196035563 8.219271674 -1.717083013

N -5.894593560 9.321853779 -1.893388648

C -5.810441356 9.865841459 0.499091146

C -3.941956380 7.640251480 2.328133423

C -2.212897455 4.916235748 1.000725462

C -2.602676548 4.309482545 -0.275252590

C -3.360543024 4.842568496 -1.331158818

C -5.018423692 7.437410941 -2.833344876

C -6.177450841 10.113965845 -0.864718119

C -4.186371257 8.554519491 4.915288872

C -5.218057560 6.569915031 -5.427757353

C -6.334602591 10.744957372 1.592003614

C -5.473539594 11.511585546 2.393903076

C -5.979846491 12.343907504 3.390146196

C -7.358208514 12.415662941 3.615573152

C -8.222114550 11.649146504 2.833743898

C -7.713130269 10.822505649 1.829451070

C -1.129553702 4.223025864 1.836222891

C -3.652523324 3.948744301 -2.525675683

C -4.819250343 3.171283015 -2.531221791

C -5.112373487 2.328070137 -3.605075089

C -4.245060137 2.254196635 -4.696102606

C -3.086329010 3.035053916 -4.704929244

C -2.793566582 3.876948236 -3.631905758

C -6.943845049 11.329178502 -1.281389806

C -6.625637513 12.622562429 -0.833921994

C -7.330405522 13.736301696 -1.291544437

C -8.366692693 13.587500271 -2.215101409

C -8.684713078 12.309496825 -2.681185455

C -7.981515593 11.197049937 -2.221781240

S -3.933402896 -1.838450596 -0.138164140

S 4.092051200 1.487237664 0.084446295

N -0.057454860 -1.831919433 -0.248562555

N -1.313350521 -2.349836828 -0.420475166

N -1.848652730 -0.126299194 0.003455420

N -2.827992331 0.620528326 0.615573478

N 0.191610683 1.894735271 -0.091953607

N 1.541142780 2.294831671 -0.078134112

N 1.817976008 0.000000000 -0.000000000

N 2.709746463 -0.976959211 0.000778650

C 0.962842942 -2.683732426 -0.301800035

C -2.213819435 -1.411179589 -0.191221598

C -2.898808004 1.898727282 0.514081298

C -2.131317500 2.840270828 -0.445486842

C -0.607346653 2.793644719 -0.610789392

C 2.337069491 1.274655227 0.002298352

C 2.328809173 -2.232548639 -0.117072711

C -3.837454781 -3.606838235 -0.598010523

C 4.168836734 3.314471489 0.028417246

C 0.691770944 -4.123963362 -0.608720741

C -0.047380715 -4.930319810 0.270343655

C -0.270960315 -6.274715906 -0.019920011

C 0.225801402 -6.831796248 -1.202220953

C 0.951383308 -6.034806839 -2.087472763

C 1.186605010 -4.690931335 -1.789689387

C -4.071058946 2.472621598 1.286247284

C 0.013417018 3.786224190 -1.553963341

C 0.039155828 5.179393107 -1.398597930

C 0.611190191 5.989667210 -2.382705717

C 1.151689532 5.429162783 -3.539977350

C 1.134577646 4.041429886 -3.705023240

C 0.581935253 3.230588881 -2.717234729

C 3.474304149 -3.193776941 -0.053087087

C 3.457178588 -4.333734497 0.766369037

C 4.568144593 -5.173372055 0.847310825

C 5.722186098 -4.889964851 0.114539672

C 5.756754051 -3.752511833 -0.695132983

C 4.646355874 -2.913324402 -0.775473558

Ni 0.000000000 0.000000000 0.000000000

H -5.267893955 8.435273746 4.828430675

H -3.917038444 9.559804556 4.587219900

H -3.876816679 8.405848523 5.954718373

H -5.603453332 6.790233828 -6.428078180

H -5.645611818 5.635860992 -5.058974625

H -4.131647780 6.469844847 -5.460072868

H -4.402516823 11.447014696 2.230397441

H -5.297457394 12.936395517 3.995430063

H -7.752505486 13.063250065 4.395228708

H -9.295744904 11.694427659 3.000490253

H -8.389441127 10.234463822 1.215874035

H -0.138476299 4.260114037 1.367848469

H -1.342500243 3.167729592 2.039197241

H -1.065900036 4.748039064 2.790394848

H -5.503981929 3.232347384 -1.689825667

H -6.018106439 1.726988890 -3.584743547

H -4.468623296 1.594311803 -5.530775693

H -2.406496158 2.991839978 -5.552738255

H -1.902452702 4.494356774 -3.654716490

H -5.815313328 12.759991675 -0.126271502

H -7.060262129 14.725716965 -0.929120424

H -8.916067764 14.456248745 -2.570628239

H -9.485648501 12.176566047 -3.405266137

H -8.220161056 10.206063058 -2.594000405

H -2.489468804 2.513307801 -1.430654584

H -3.404688429 -3.731258185 -1.592111605

H -3.242718061 -4.170317988 0.122156402

H -4.870581047 -3.966917305 -0.593406917

H 5.232891775 3.565033329 0.065185291

H 3.724548121 3.694481036 -0.892757854

H 3.652321585 3.752438730 0.884057952

H -0.446689985 -4.495504649 1.181158457

H -0.837911877 -6.888650554 0.675638986

H 0.047084718 -7.879770102 -1.429704152

H 1.341015984 -6.457293658 -3.010146330

H 1.762760989 -4.076348625 -2.475282732

H -3.759513543 3.203386811 2.038392035

H -4.767539411 2.993650650 0.621290446

H -4.589141108 1.648115212 1.780372198

H -0.369434725 5.641809617 -0.510828099

H 0.621751414 7.066675641 -2.240334702

H 1.582640864 6.067177063 -4.307418312

H 1.551522342 3.589118780 -4.601163686

H 0.570869308 2.152520210 -2.848825770

H 2.572132338 -4.562711511 1.350278664

H 4.532017482 -6.048193827 1.491982518

H 6.586734438 -5.546326128 0.176938798

H 6.650819092 -3.516840309 -1.267465432

H 4.676842065 -2.022673790 -1.394957715

**Table S11**. Cartesian coordinates of optimized geometry **d2**.

opt b3lyp/6-31g* freq=OK charge=-1 multiplicity =1

127

Ni -4.484543809 4.081636211 4.800847363

S -6.281487676 0.260806888 3.742879715

S -2.628809480 7.592004441 6.570519707

N -6.181481763 3.762909567 5.432144619

N -6.769422320 2.610638290 4.963776703

N -4.682652028 2.434773917 3.963281752

N -3.700324689 1.519176445 3.671694714

N -2.804582218 4.534795024 4.103228268

N -2.281728319 5.691281422 4.664010161

N -4.297938986 5.589427832 5.781244786

N -5.100880545 6.182019115 6.636736721

C -6.918509990 4.565464339 6.210917082

C -5.886101485 1.911054608 4.277498101

C -2.681508292 1.862399386 2.951419481

C -2.503130268 3.111118301 2.165381530

C -2.482159587 4.358063127 2.758424418

C -3.063214259 6.178359102 5.576639942

C -6.333532189 5.722751327 6.839201056

C -7.987922949 0.119073740 4.382623387

C -0.980779484 7.938828957 5.863073494

C -8.374938191 4.268107286 6.392089660

C -8.821690391 3.083346236 6.999037943

C -10.183469144 2.838862381 7.167860989

C -11.128006992 3.767051299 6.719500426

C -10.696364325 4.942325735 6.104409494

C -9.331415055 5.190166943 5.945190832

C -1.578184865 0.831114534 2.949501140

C -2.466536732 5.632167894 1.946133036

C -3.538575084 5.850547167 1.062343127

C -3.674841245 7.054637868 0.367654531

C -2.746498634 8.078211763 0.554992362

C -1.687058388 7.881642798 1.443271586

C -1.549880512 6.677528319 2.133908847

C -7.080809615 6.547331290 7.838895742

C -7.832802681 5.985284435 8.884839360

C -8.468665269 6.791341352 9.829430066

C -8.362903801 8.181717060 9.759669097

C -7.605702422 8.755548781 8.735445746

C -6.973179877 7.949339033 7.790432481

S -3.791500620 -1.871138933 -1.044884374

S 4.061812971 1.460268906 -0.466339964

N -0.019081240 -1.852124345 -0.137860346

N -1.219425870 -2.395356002 -0.503783139

N -1.829799316 -0.162017970 -0.295599696

N -2.926632697 0.635852110 -0.381500275

N 0.200826436 1.910118271 0.085158795

N 1.529070463 2.288339877 -0.203776681

N 1.824446571 0.000000000 -0.000000000

N 2.727397287 -0.958447250 0.131713500

C 0.995332021 -2.695639018 0.024067540

C -2.134951343 -1.450240765 -0.579427676

C -3.024121817 1.846967202 0.022077116

C -2.063888614 2.844015606 0.684863100

C -0.569975061 2.925514122 0.380927192

C 2.322767574 1.263353587 -0.200876952

C 2.351357057 -2.216892289 0.218413682

C -3.581486571 -3.667593031 -1.331465148

C 4.120169440 3.264405459 -0.762946068

C 0.738058458 -4.163500012 -0.114531194

C -0.102032520 -4.831124480 0.790074538

C -0.322176457 -6.201790143 0.672146541

C 0.279684089 -6.924618776 -0.362440354

C 1.105258682 -6.266275056 -1.273868928

C 1.336423737 -4.895011006 -1.147789325

C -4.416820398 2.425183280 -0.153998790

C 0.061468753 4.285606099 0.372915350

C 0.979471121 4.657364904 1.364706590

C 1.670813269 5.862055516 1.264378488

C 1.459782462 6.704245576 0.168314472

C 0.535978600 6.345103421 -0.812664061

C -0.165414834 5.142573889 -0.708589881

C 3.486418374 -3.153492392 0.484461891

C 3.411833019 -4.157027739 1.463386456

C 4.508805971 -4.975608467 1.732405734

C 5.704320848 -4.806489256 1.031643505

C 5.794487849 -3.804223792 0.062883221

C 4.698710620 -2.985367918 -0.205401999

Ni 0.000000000 0.000000000 0.000000000

H -8.626321484 0.903167486 3.972334429

H -8.005022145 0.184265835 5.472005291

H -8.346142959 -0.864191972 4.062297336

H -0.568895006 8.768759481 6.445467585

H -1.060586453 8.226486984 4.812617009

H -0.331310137 7.064867126 5.946293865

H -8.091588472 2.355020034 7.335233495

H -10.509601632 1.918588416 7.647336278

H -12.190407185 3.573325829 6.848468765

H -11.420706302 5.671724952 5.749524645

H -8.998301795 6.110824072 5.474953920

H -0.747955009 1.189073846 3.576361987

H -1.164273963 0.632068743 1.960546314

H -1.952562512 -0.104636091 3.373523718

H -4.292762938 5.078696687 0.949072745

H -4.520122737 7.194883852 -0.302189623

H -2.851980183 9.021753867 0.024486966

H -0.958399850 8.672896843 1.603145864

H -0.732053005 6.544339916 2.827113401

H -7.915228031 4.906891863 8.965103274

H -9.041757681 6.327090783 10.629083668

H -8.859585434 8.809352244 10.496176450

H -7.507889322 9.837099498 8.669633498

H -6.374782115 8.395318860 7.002678265

H -2.382176994 3.777721124 0.196131366

H -2.852730413 -3.861138356 -2.120450262

H -3.265702555 -4.179538833 -0.421302060

H -4.568102080 -4.028324179 -1.637803198

H 5.174163742 3.497916621 -0.939316195

H 3.525912633 3.534583762 -1.637437031

H 3.752086435 3.817232652 0.101999777

H -0.583692827 -4.268067995 1.583615993

H -0.968420644 -6.706234359 1.386078568

H 0.104320849 -7.993473550 -0.455819268

H 1.576041023 -6.818564469 -2.083228459

H 1.988909745 -4.388058069 -1.852559482

H -4.836340791 2.723339768 0.814060222

H -4.402279476 3.315304486 -0.797827300

H -5.066890167 1.671736656 -0.601475852

H 1.156470684 3.992763744 2.204450049

H 2.376587606 6.143418170 2.041899878

H 2.005571979 7.640896868 0.087111601

H 0.355582910 7.000269996 -1.660559713

H -0.873457239 4.860810035 -1.483415886

H 2.492468929 -4.294860007 2.022770100

H 4.428513656 -5.743463559 2.497898287

H 6.558008340 -5.446415176 1.240884254

H 6.721094624 -3.658981958 -0.487399453

H 4.771417685 -2.198939891 -0.949910951

**Table S12**. Cartesian coordinates of optimized geometry **d3**.

opt b3lyp/6-31g* freq=OK charge=-1 multiplicity =1

127

Ni -3.525576115 3.828687810 4.719403101

S -6.383368586 4.822984910 1.580691906

S -1.470515403 2.555427036 8.342371968

N -4.474982972 5.390672025 4.919628564

N -5.251614167 5.752843495 3.835689920

N -4.411653240 3.724091947 3.078428848

N -4.811334742 2.530984817 2.497254458

N -2.473759749 2.292491333 4.554106147

N -1.816449420 1.935135164 5.729724166

N -2.849312431 3.891330964 6.393748405

N -3.012107510 4.764446994 7.360940599

C -4.408702834 6.239942187 5.949140662

C -5.258036514 4.766827509 2.959699177

C -3.893487928 1.656921668 2.244484812

C -2.407105860 1.906929929 2.203320748

C -1.736976713 2.107082630 3.391040240

C -2.084564549 2.765684806 6.684468102

C -3.717989969 5.875634482 7.164589295

C -7.201716295 6.427769329 1.887631521

C -0.525171437 1.011799262 8.086679628

C -5.035085922 7.594249504 5.819183548

C -6.416875782 7.757751461 5.632178344

C -6.975439923 9.030324085 5.525362170

C -6.161773499 10.165501142 5.587912637

C -4.785895965 10.014720563 5.761911771

C -4.229370034 8.739463221 5.879286642

C -4.386932696 0.259173296 1.974853780

C -0.249427389 2.149214827 3.554452920

C 0.355123409 3.220501105 4.237536533

C 1.722994905 3.221162203 4.499185980

C 2.519231325 2.146617231 4.086240895

C 1.930779792 1.070540481 3.419986217

C 0.555782157 1.070684565 3.168270270

C -3.752475646 6.738892235 8.386013885

C -4.931651576 7.324911837 8.877701209

C -4.927447449 8.076614700 10.052934706

C -3.745760077 8.255941386 10.774480482

C -2.568306297 7.666245306 10.308258126

C -2.573141659 6.918089533 9.132127582

S -3.667889164 -1.807704390 -1.449903746

S 4.047648878 1.436459559 -0.577953311

N -0.046713168 -1.847313900 -0.063337961

N -1.232086196 -2.386131107 -0.491907908

N -1.803937597 -0.134025343 -0.416907241

N -2.719812749 0.770631850 -0.886159953

N 0.213978512 1.882644121 0.060405390

N 1.517668036 2.281449639 -0.280089456

N 1.817627519 0.000000000 0.000000000

N 2.708318225 -0.956624735 0.201248162

C 0.955370992 -2.687333135 0.172354707

C -2.097704762 -1.416243140 -0.726220676

C -2.807139855 1.945267772 -0.375197528

C -2.037299225 2.538023478 0.823272198

C -0.577438955 2.827262777 0.485721888

C 2.316917291 1.257679412 -0.260229860

C 2.312580742 -2.203151618 0.370424588

C -3.498784916 -3.623241158 -1.602163456

C 4.103765549 3.224899288 -0.956930698

C 0.693550986 -4.157978350 0.091617610

C -0.205563069 -4.771697475 0.977680194

C -0.436549431 -6.144178983 0.914490966

C 0.213489216 -6.923568025 -0.047181246

C 1.097809792 -6.319504085 -0.941176141

C 1.339442810 -4.946343021 -0.869237752

C -3.814224629 2.858198968 -1.031275623

C -0.127098500 4.245552791 0.610236811

C 1.057257645 4.591455879 1.284417653

C 1.432163435 5.925989706 1.417446290

C 0.650022353 6.941657640 0.861339266

C -0.523016532 6.611994495 0.183619819

C -0.914704943 5.277554257 0.072000570

C 3.428260332 -3.130154818 0.732070123

C 3.307202629 -4.070466961 1.767745946

C 4.384184904 -4.880985612 2.126904753

C 5.606059317 -4.766849286 1.461419478

C 5.742348330 -3.827505829 0.436423084

C 4.666633166 -3.016803097 0.077872510

Ni 0.000000000 0.000000000 0.000000000

H -6.473238340 7.239636528 1.910114038

H -7.741868661 6.411741426 2.835672700

H -7.902904479 6.571324761 1.059815880

H -0.094943462 0.754859943 9.059546723

H 0.268276469 1.160788044 7.351878452

H -1.180027154 0.209313853 7.741461830

H -7.048535765 6.878097065 5.569795886

H -8.049281009 9.137058121 5.388929775

H -6.598404095 11.157787381 5.501157558

H -4.141823118 10.889534505 5.811134440

H -3.159165615 8.625837882 6.025794333

H -4.136360364 -0.367579353 2.842880890

H -3.919562514 -0.199193629 1.102485140

H -5.474746178 0.265497051 1.856667388

H -0.264440538 4.048694595 4.568366471

H 2.169061666 4.059824364 5.028813748

H 3.586999888 2.143530630 4.292413189

H 2.536551387 0.225294769 3.103982074

H 0.090644010 0.212405245 2.693951936

H -5.863388135 7.184986257 8.340670474

H -5.856653873 8.515437272 10.409829261

H -3.743456277 8.842521795 11.690408059

H -1.639846541 7.790115905 10.861583949

H -1.660693261 6.449265830 8.778224536

H -2.474392375 3.538055003 0.927122673

H -2.643850566 -3.885666519 -2.227128682

H -3.382246860 -4.091912419 -0.623855669

H -4.426644164 -3.964186323 -2.071132123

H 5.150224666 3.443577760 -1.188131659

H 3.473992085 3.459683670 -1.816756478

H 3.778080033 3.818089873 -0.101774135

H -0.723924659 -4.165087108 1.713875303

H -1.128691641 -6.605919394 1.614188209

H 0.030048087 -7.993973685 -0.097922851

H 1.606165757 -6.915954163 -1.694553337

H 2.036874972 -4.481750177 -1.559872891

H -4.522278142 3.258751998 -0.296887445

H -3.318088553 3.717380362 -1.505497207

H -4.360190811 2.310836365 -1.803124976

H 1.665166815 3.814765180 1.726799346

H 2.337567390 6.172772600 1.965893459

H 0.948438544 7.981826667 0.964811889

H -1.144684256 7.390506777 -0.249954433

H -1.832416580 5.042757212 -0.456685745

H 2.366673906 -4.164389139 2.300671141

H 4.267477768 -5.598698501 2.935170524

H 6.444359669 -5.400189250 1.741066904

H 6.689766766 -3.725444902 -0.087567406

H 4.775065673 -2.278858166 -0.710585382

**Table S13**. Cartesian coordinates of optimized geometry **e**.

opt b3lyp/6-31g* freq=OK charge=-1 multiplicity =1

127

Ni -3.107577424 4.651549209 4.362968624

S -5.287944141 6.481672473 1.057028444

S -1.727892995 2.893056506 8.102515157

N -3.436883651 6.457895314 4.478199141

N -3.991847780 7.030972804 3.349761429

N -3.944194301 4.802816239 2.700762019

N -4.728556674 3.822743561 2.116530763

N -2.635168816 2.843222829 4.284176709

N -2.199365892 2.324133171 5.498655967

N -2.497790082 4.543738044 6.062022415

N -2.366429576 5.457126716 6.995917976

C -3.101793820 7.270528603 5.484709395

C -4.332872348 6.078355927 2.504885055

C -4.178161157 2.672516737 1.914758951

C -2.709136840 2.355074882 1.953214775

C -2.046395673 2.290424823 3.160596229

C -2.184907835 3.235807973 6.416297368

C -2.625200005 6.736991935 6.739402678

C -5.490432526 8.283136277 1.288812092

C -1.390751552 1.104079942 7.934431335

C -3.193441649 8.752426341 5.287540895

C -4.415776673 9.392988290 5.029313528

C -4.474042313 10.775580954 4.862556946

C -3.308851319 11.544800972 4.935086108

C -2.086956119 10.917720061 5.179581343

C -2.031935655 9.533778143 5.356983523

C -5.123698844 1.544334433 1.578432895

C -0.731061849 1.608798429 3.364125647

C 0.324320197 2.248999049 4.037055477

C 1.506087634 1.568754843 4.323226863

C 1.645191031 0.221571981 3.969835052

C 0.594489155 -0.431464009 3.327284804

C -0.582625665 0.258810925 3.021035932

C -2.391186817 7.605464854 7.934705117

C -3.299091816 8.593202193 8.353982241

C -3.067948230 9.341612924 9.508402895

C -1.927032353 9.117176641 10.281132923

C -1.023020315 8.127635782 9.887296387

C -1.253350339 7.382741879 8.731840530

S -3.776944802 -1.889807757 -1.050816121

S 4.074437523 1.451427160 -0.435203043

N -0.057368681 -1.853111589 0.048200386

N -1.283145480 -2.409038845 -0.216695652

N -1.826355321 -0.151882764 -0.345409110

N -2.698120730 0.702854652 -0.975304947

N 0.213068059 1.892943695 0.046247850

N 1.538571962 2.291532885 -0.193141770

N 1.819758010 -0.000000000 0.000000000

N 2.710247116 -0.962186611 0.160276087

C 0.957164596 -2.692102492 0.231102945

C -2.152454698 -1.453802121 -0.489081549

C -2.763218704 1.935187327 -0.627158342

C -2.090943693 2.651311545 0.554850908

C -0.588018166 2.845660889 0.443936632

C 2.331363049 1.264337592 -0.190669198

C 2.324373449 -2.210922877 0.333049865

C -3.650651113 -3.714173517 -1.004551980

C 4.142719897 3.254796674 -0.735738019

C 0.685037076 -4.164097312 0.215317181

C -0.121597345 -4.756758326 1.199078881

C -0.355807137 -6.130175639 1.191128736

C 0.197786844 -6.932601402 0.188918993

C 0.988580566 -6.349976315 -0.801594819

C 1.233669193 -4.975480619 -0.785824282

C -3.675897848 2.797794196 -1.466234562

C -0.085284913 4.224268705 0.732213582

C 1.005033674 4.439529582 1.594515825

C 1.431197778 5.730646561 1.893468103

C 0.797961432 6.835982492 1.317914800

C -0.270945499 6.637231979 0.445875337

C -0.713988148 5.344537436 0.163403733

C 3.465542295 -3.139186686 0.605184297

C 3.431888879 -4.085117532 1.642448101

C 4.537206926 -4.892514954 1.911212500

C 5.702329448 -4.769970996 1.152031569

C 5.752824738 -3.825285620 0.124319130

C 4.648289414 -3.018264149 -0.143936343

Ni 0.000000000 0.000000000 0.000000000

H -4.521126617 8.779652391 1.353129076

H -6.056757496 8.495840436 2.197135219

H -6.041516185 8.639747104 0.413172737

H -1.106201724 0.754610142 8.931668311

H -0.578010419 0.926552287 7.227547801

H -2.280402339 0.573071379 7.590065098

H -5.319235724 8.796830987 4.958936732

H -5.431671486 11.254736485 4.671515554

H -3.355226972 12.623156306 4.801683939

H -1.173208297 11.504502283 5.237685581

H -1.080724950 9.049779534 5.558529049

H -5.156868785 0.847530341 2.427852982

H -4.802213882 0.963888028 0.708535343

H -6.131494396 1.938920638 1.415586232

H 0.203115005 3.284250431 4.340407486

H 2.315219318 2.083733005 4.835960077

H 2.562503447 -0.313841544 4.201086914

H 0.688482486 -1.480770995 3.061259336

H -1.416277006 -0.254808929 2.553946404

H -4.199437041 8.772744030 7.776708466

H -3.790545154 10.097221798 9.808856088

H -1.747595704 9.701448374 11.180793823

H -0.132220818 7.934808271 10.481302475

H -0.557389549 6.605068234 8.434392811

H -2.439252414 3.683410603 0.427011774

H -2.841591991 -4.065071150 -1.646849494

H -3.479624577 -4.072441199 0.011799469

H -4.612047967 -4.084014805 -1.373333371

H 5.199590859 3.485891277 -0.897299405

H 3.562264047 3.524597881 -1.619775061

H 3.764780174 3.810861360 0.123024398

H -0.568554274 -4.132931077 1.967079045

H -0.975809348 -6.574737913 1.965672986

H 0.011839514 -8.003756979 0.181480368

H 1.421517152 -6.964102031 -1.587224377

H 1.859508836 -4.527511461 -1.551972174

H -4.418640841 3.311874733 -0.844215479

H -3.098912992 3.578335244 -1.983640493

H -4.183184005 2.187088207 -2.216638872

H 1.496047812 3.589660356 2.049454947

H 2.257740815 5.874368089 2.584651024

H 1.131097508 7.843032191 1.554737883

H -0.774746882 7.485873118 -0.007918678

H -1.545866713 5.216549221 -0.522143232

H 2.537181042 -4.187768878 2.247537632

H 4.487510029 -5.614733970 2.722469253

H 6.562731346 -5.400893640 1.361308153

H 6.655307055 -3.715983699 -0.472511205

H 4.691184063 -2.276061258 -0.934667481

**Table S14**. Cartesian coordinates of optimized geometry **g**.

opt b3lyp/6-31g* freq=OK charge=-1 multiplicity =1

127

Ni -2.764492130 4.519215980 4.531806170

S -5.431099528 6.279004993 1.561586951

S -0.783592617 2.806591350 8.015922683

N -3.137160104 6.313950323 4.703932887

N -3.854725680 6.871316334 3.661721093

N -3.811584838 4.650079895 2.991616461

N -4.606701199 3.645559097 2.467444073

N -2.233344495 2.733330368 4.368023629

N -1.601503012 2.232110605 5.501286430

N -1.919328155 4.430441448 6.130179536

N -1.702036442 5.344014163 7.047059266

C -2.696733745 7.134885853 5.663181047

C -4.272517115 5.909848890 2.860598920

C -4.030380746 2.531213846 2.162840811

C -2.553672295 2.276755568 2.043301873

C -1.760712931 2.227592822 3.167679966

C -1.497098010 3.136990393 6.418877614

C -2.041438538 6.613920634 6.839696108

C -5.650115132 8.074771636 1.820039439

C -0.364680604 1.044673541 7.766664756

C -2.850368434 8.614109918 5.485483369

C -4.110251555 9.225044988 5.382530301

C -4.221345592 10.605652656 5.226978952

C -3.074843255 11.402553320 5.155127906

C -1.817415628 10.805369521 5.245335869

C -1.707982063 9.423350125 5.412453685

C -4.957242874 1.375767717 1.870087231

C -0.388364481 1.627358451 3.216696126

C 0.741036933 2.394731868 3.539127357

C 1.985220345 1.790118359 3.712463334

C 2.114476937 0.401835834 3.604083391

C 0.993149491 -0.371655842 3.303387749

C -0.245897945 0.238926216 3.093678410

C -1.689099971 7.485859061 8.002936835

C -2.571978577 8.438830343 8.539388035

C -2.224504589 9.192453236 9.660772113

C -0.988167771 9.007587330 10.282419716

C -0.105933905 8.052524427 9.771387093

C -0.452747866 7.302401657 8.648879402

S -3.776845049 -1.905422745 -1.044059923

S 4.092757637 1.466215234 -0.215091637

N -0.048215972 -1.857209510 0.022241508

N -1.270866804 -2.417525574 -0.247545575

N -1.830404570 -0.161967734 -0.338205312

N -2.746521818 0.683644620 -0.912744419

N 0.210769115 1.899016493 0.038177243

N 1.546277479 2.297118292 -0.134637978

N 1.820006344 0.000000000 0.000000000

N 2.711911575 -0.961240007 0.160026247

C 0.966603358 -2.695960415 0.196197231

C -2.148228125 -1.464638535 -0.495840466

C -2.825678824 1.908969672 -0.546013970

C -2.093721228 2.628245044 0.597790972

C -0.610394696 2.867863169 0.338790490

C 2.337581406 1.269568696 -0.115697123

C 2.332480156 -2.213321314 0.310371934

C -3.636819803 -3.729438547 -1.016798394

C 4.184061848 3.288141405 -0.336489503

C 0.698626843 -4.168366706 0.162053188

C -0.105237203 -4.776210834 1.138739197

C -0.334481549 -6.150250504 1.113363155

C 0.221245682 -6.937816867 0.100552076

C 1.009622505 -6.339884077 -0.882702022

C 1.250091311 -4.964850848 -0.849303528

C -3.810770701 2.760234727 -1.309998550

C -0.203429372 4.307074780 0.370753997

C -0.664631761 5.133928859 1.411381774

C -0.375390173 6.498552041 1.416977367

C 0.355792522 7.066786474 0.373356747

C 0.798952248 6.258671344 -0.677041148

C 0.530881886 4.891380896 -0.677012538

C 3.476009123 -3.140463055 0.575996792

C 3.440687215 -4.102264207 1.598609308

C 4.548928788 -4.907119786 1.862872790

C 5.718448827 -4.766562790 1.113611147

C 5.770442544 -3.806382937 0.100506982

C 4.663050085 -3.001753601 -0.163153573

Ni 0.000000000 0.000000000 0.000000000

H -4.697456418 8.600067741 1.737288329

H -6.081790440 8.276646865 2.801791894

H -6.334890259 8.409589483 1.034890400

H 0.087088309 0.705686137 8.703954276

H 0.342744601 0.926961750 6.943561472

H -1.260784630 0.460242185 7.548777030

H -5.001067850 8.607687024 5.423766352

H -5.206349082 11.061640343 5.157224467

H -3.163181400 12.479220348 5.029975514

H -0.917741524 11.413758861 5.190997269

H -0.727606600 8.963192653 5.496553077

H -4.878260704 0.643578413 2.685937022

H -4.697937682 0.847104202 0.948000129

H -5.991395067 1.730525907 1.821491605

H 0.637555188 3.468881167 3.662050693

H 2.853567313 2.400606312 3.947911280

H 3.082130105 -0.071089454 3.749056550

H 1.083989490 -1.451648475 3.220869738

H -1.125684481 -0.360025506 2.879004443

H -3.543545524 8.586700172 8.080576638

H -2.930018145 9.920669684 10.054818972

H -0.717988720 9.595815047 11.156435762

H 0.858786502 7.890910163 10.247453803

H 0.227674435 6.551298353 8.260982874

H -2.491869034 3.645276580 0.505630728

H -2.835634053 -4.069410716 -1.674658846

H -3.447779294 -4.096207533 -0.006674335

H -4.601362844 -4.102366526 -1.374254109

H 5.250303188 3.531720488 -0.322010335

H 3.735035303 3.645774282 -1.264847848

H 3.678795441 3.755482215 0.509890204

H -0.553304863 -4.163513977 1.914931653

H -0.952410073 -6.606962892 1.882494066

H 0.038952570 -8.009424433 0.079432650

H 1.444380394 -6.942431630 -1.676245476

H 1.874218668 -4.504997956 -1.609835701

H -4.541030189 3.219262904 -0.631800658

H -3.292051532 3.585118132 -1.818843609

H -4.331744973 2.156212326 -2.056432049

H -1.259313143 4.722026924 2.220199000

H -0.746156772 7.112877461 2.232627705

H 0.570250761 8.132618286 0.371905676

H 1.355617891 6.694046300 -1.503538815

H 0.881589067 4.271257708 -1.492533169

H 2.542541844 -4.219385172 2.195873886

H 4.498040369 -5.641517693 2.663030172

H 6.581056878 -5.395582747 1.319427681

H 6.676319041 -3.682961338 -0.488351633

H 4.707003686 -2.247519934 -0.942340972

**Table S15**. Cartesian coordinates of optimized geometry **s**.

opt b3lyp/6-31g* freq=OK charge=-1 multiplicity =1

127

Ni -2.929650000 3.884960000 4.977420002

S -5.998070002 5.412300002 2.279190000

S -0.498300000 2.303740000 8.230860002

N -3.505360000 5.610370002 5.259900002

N -4.358710002 6.114060002 4.295350002

N -4.093900000 3.943060000 3.519230000

N -4.799190002 2.868220000 3.005530000

N -2.194360000 2.189170000 4.691770002

N -1.424510000 1.728000000 5.754140002

N -1.964660000 3.841720000 6.508660002

N -1.795000000 4.738630002 7.451550002

C -3.103530000 6.441400002 6.227270002

C -4.712800002 5.139880002 3.479270000

C -4.117550000 1.845830000 2.609820000

C -2.633990000 1.778710000 2.377110000

C -1.758700000 1.786250000 3.440270000

C -1.364830000 2.602270000 6.704880002

C -2.304110000 5.962220002 7.330000002

C -6.406090002 7.158940002 2.629060000

C 0.115260000 0.619940000 7.869590002

C -3.455270000 7.894290002 6.133210002

C -4.786580002 8.339860002 6.147930002

C -5.083150002 9.699380002 6.068690002

C -4.055440000 10.640730002 5.957050002

C -2.729320000 10.208910002 5.930360002

C -2.433360000 8.847200002 6.021070002

C -4.918590002 0.597130000 2.328260000

C -0.325670000 1.353790000 3.365390000

C 0.726740000 2.238460000 3.645070000

C 2.042610000 1.781780000 3.703680000

C 2.325380000 0.424290000 3.521520000

C 1.283320000 -0.466320000 3.262590000

C -0.030920000 -0.001680000 3.167840000

C -1.978050000 6.825000002 8.507630002

C -2.931250000 7.632730002 9.151420002

C -2.597890000 8.379350002 10.281730002

C -1.304490000 8.331910002 10.805210002

C -0.349770000 7.521000002 10.186770002

C -0.683000000 6.777970002 9.055450002

S -3.563020000 -2.406840000 -0.812490000

S 3.878980000 1.884870000 -0.364510000

N 0.200670000 -1.934980000 -0.004130000

N -0.961320000 -2.632470000 -0.215540000

N -1.795990000 -0.462470000 -0.161200000

N -2.848900000 0.283320000 -0.629460000

N 0.001860000 1.820570000 0.170180000

N 1.263100000 2.388290000 -0.073150000

N 1.822500000 0.139590000 -0.069340000

N 2.833980000 -0.708520000 -0.014540000

C 1.319780000 -2.647000000 0.061450000

C -1.964010000 -1.787590000 -0.357760000

C -3.049050000 1.474460000 -0.201520000

C -2.326850000 2.236750000 0.920490000

C -0.906210000 2.667330000 0.573110000

C 2.172510000 1.467400000 -0.157180000

C 2.621620000 -2.002900000 0.102920000

C -3.200440000 -4.198940002 -0.879040000

C 3.738650000 3.707300000 -0.405460000

C 1.230110000 -4.139050000 -0.019820000

C 0.581540000 -4.876000002 0.983110000

C 0.518860000 -6.266090002 0.913010000

C 1.087990000 -6.942390002 -0.170320000

C 1.721960000 -6.216960002 -1.179120000

C 1.796170000 -4.824770002 -1.101810000

C -4.184080000 2.224620000 -0.855740000

C -0.674580000 4.143570000 0.647030000

C -0.096160000 4.854530002 -0.420050000

C 0.003820000 6.243470002 -0.373910000

C -0.455120000 6.949330002 0.741380000

C -1.033280000 6.254980002 1.804490000

C -1.154730000 4.866210002 1.754430000

C 3.885810000 -2.790140000 0.244040000

C 4.044320000 -3.788040000 1.219270000

C 5.258540002 -4.458750002 1.366360000

C 6.342670002 -4.144910000 0.544710000

C 6.201590002 -3.146900000 -0.422300000

C 4.988260002 -2.476410000 -0.568910000

Ni 0.021170000 -0.086810000 0.057550000

H -5.530520002 7.797260002 2.501240000

H -6.782710002 7.274230002 3.646750000

H -7.182680002 7.435660002 1.909450000

H 0.674540000 0.305140000 8.755930002

H 0.767980000 0.624460000 6.994510002

H -0.715590000 -0.064490000 7.687160002

H -5.586340002 7.610730002 6.220290002

H -6.120420002 10.025950002 6.089850002

H -4.288620002 11.700940002 5.891620002

H -1.920640000 10.930750002 5.843910002

H -1.399060000 8.515360002 6.014180002

H -4.690300002 -0.151350000 3.100020000

H -4.668730002 0.141050000 1.365660000

H -5.988540002 0.823220000 2.370140000

H 0.507150000 3.286660000 3.825830000

H 2.848420000 2.482730000 3.907340000

H 3.349860000 0.066010000 3.577130000

H 1.493720000 -1.523590000 3.123540000

H -0.848010000 -0.693240000 2.986300000

H -3.945780000 7.671310002 8.769980002

H -3.357990000 8.993230002 10.759970002

H -1.045440000 8.914640002 11.686260002

H 0.661010000 7.467130002 10.585340002

H 0.055830000 6.138190002 8.583860002

H -2.850050000 3.199700000 0.901450000

H -2.413430000 -4.411400002 -1.604100000

H -2.895590000 -4.576960002 0.098100000

H -4.135710000 -4.675550002 -1.187760000

H 4.765530002 4.081430000 -0.449700000

H 3.184370000 4.041700000 -1.284260000

H 3.241630000 4.074710000 0.493480000

H 0.122330000 -4.351360002 1.815310000

H 0.020350000 -6.822760002 1.702680000

H 1.035640000 -8.026900002 -0.226040000

H 2.165420000 -6.732560002 -2.027240000

H 2.300920000 -4.264490002 -1.883300000

H -4.682200002 1.588630000 -1.591130000

H -4.912030002 2.564400000 -0.108380000

H -3.808180000 3.125600000 -1.361050000

H 0.265620000 4.314680002 -1.286270000

H 0.441090000 6.775990002 -1.215190000

H -0.372130000 8.032820002 0.775560000

H -1.414670000 6.786780002 2.671610000

H -1.634400000 4.353010002 2.581430000

H 3.214610000 -4.039080000 1.871690000

H 5.357300002 -5.224500002 2.131900000

H 7.288070002 -4.669530002 0.659190000

H 7.038880002 -2.889040000 -1.066550000

H 4.881700002 -1.692790000 -1.312230000
